# Gene and transposable element methylation in great tit (*Parus major*) brain and blood

**DOI:** 10.1186/s12864-016-2653-y

**Published:** 2016-05-04

**Authors:** Martijn F. L. Derks, Kyle M. Schachtschneider, Ole Madsen, Elio Schijlen, Koen J. F. Verhoeven, Kees van Oers

**Affiliations:** Department of Animal Ecology, Netherlands Institute of Ecology (NIOO-KNAW), Wageningen, The Netherlands; Bioinformatics Group, Wageningen University, Wageningen, The Netherlands; Animal Breeding and Genomics Centre, Wageningen University, Wageningen, The Netherlands; Department of Animal Sciences, University of Illinois, Urbana-Champaign, USA; PRI Bioscience, Plant Research International, Wageningen UR, Wageningen, The Netherlands; Department of Terrestrial Ecology, Netherlands Institute of Ecology (NIOO-KNAW), Wageningen, The Netherlands

**Keywords:** Whole genome bisulfite sequencing, Parus major, Differential methylation, CGIs, Brain methylation, Gene feature methylation, non-CpG methylation, TE methylation

## Abstract

**Background:**

Studies on vertebrate DNA methylomes have revealed a regulatory role of tissue specific DNA methylation in relation to gene expression. However, it is not well known how tissue-specific methylation varies between different functional and structural components of genes and genomes. Using whole-genome bisulfite sequencing data we here describe both CpG and non-CpG methylation profiles of whole blood and brain tissue in relation to gene features, CpG-islands (CGIs), transposable elements (TE), and their functional roles in an ecological model species, the great tit (*Parus major*).

**Results:**

We show that hypomethylation at the transcription start site (TSS) is enriched in genes with functional classes that relate directly to processes specific to each tissue type. We find that 6877 (~21 %) of the CGIs are differentially methylated between blood and brain, of which 1186 and 2055 are annotated to promoter and intragenic regions, respectively. We observe that CGI methylation in promoter regions is more conserved between tissues compared to CGI methylation in intra and inter-genic regions. Differentially methylated CGIs in promoter and intragenic regions are overrepresented in genomic loci linked to development, suggesting a distinct role for CGI methylation in regulating expression during development. Additionally, we find significant non-CpG methylation in brain but not in blood with a strong preference for methylation at CpA dinucleotide sites. Finally, CpG hypermethylation of TEs is significantly stronger in brain compared to blood, but does not correlate with TE activity. Surprisingly, TEs showed significant hypomethylation in non-CpG contexts which was negatively correlated with TE expression.

**Conclusion:**

The discovery that TSS methylation levels are directly linked to functional classes related to each tissue provides new insights in the regulatory role of DNA-methylation patterns. The dominant sequence motifs for brain non-CpG methylation, similar to those found in mammals, suggests that a conserved non-CpG regulatory mechanism was already present in the amniote ancestor. The negative correlation between brain non-CpG methylation and TE activity (not found for CpG methylation) suggests that non-CpG is the dominant regulatory form of methylation in TE silencing.

**Electronic supplementary material:**

The online version of this article (doi:10.1186/s12864-016-2653-y) contains supplementary material, which is available to authorized users.

## Background

DNA methylation is the addition of a methyl (-CH3) group to the 5’ carbon site of cytosines catalyzed by DNA-methyltransferases, which occurs mainly at CpG sites in animals [1]. It is involved in many biological processes including modulation of gene expression. The majority of methylation studies have been conducted on either humans or model species for human diseases, and studies on ecological model systems are rare. Moreover, studies on such systems, where reference genomes are often lacking, mainly use targeted methods such as MS-AFLP or RRBS that rely heavily on assumptions from human and rodent studies and provide only limited functional insights [2]. We assessed whole genome DNA methylation in the great tit, a model organism for ecological and evolutionary studies on e.g. the effects of climate change on natural populations [3], the allocation of resources to breeding [4], the impact of variation in personality traits on other life history characters [5] and one of the first in the class of Aves to study the methylome [6]. Whole methylome information will give us detailed insight into processes related to gene expression, silencing and tissue specialization, and allows us to better predict what variation is important for answering ecologically relevant questions.

Previous studies on DNA methylation in animals have focused mainly on CGIs, which are often found near the TSS and are usually hypomethylated [7]. Methylation in promoter regions is often found to be negatively-correlated with gene expression, becoming less accessible for transcription factors or RNA-polymerases [8]. However, not all functions and mechanisms of DNA methylation are well understood. For instance, a positive correlation is observed between gene expression and gene body (GB) methylation in many mammalian tissues [9]. In contrary, a slightly negative correlation was found in brain neural tissue [6, 10]. Previous studies have demonstrated that GB methylation may have a role in splicing [11, 12]. However, recent evidence contradicts this [13]. Other studies show that it could prevent interruptive transposable element (TE) insertions [14] and DNA methylation is known to suppress the activity of TEs in both plants and animals [15]. However, the underlying mechanisms of TE silencing differ between plants and animals [16]. In plants this involves small RNA-guided DNA methylation of cytosines resulting in a specific signature of dense methylation in all sequence contexts (CpG and non-CpG) [17], whereas a similar system in mammals is unknown, and DNA methylation in TEs and genes is almost exclusively found in a CpG context.

The methylation landscape can vary between tissue types, playing a large role in for example gene regulation [18]. Previous studies have identified differentially methylated regions across tissues [19–21]. However, these studies were mainly conducted in mammals and did not focus specifically on gene features, but rather on differentially methylated regions throughout the genome. The observation that DNA methylation negatively correlates with gene expression suggests that hypomethylation occurs in gene promoter regions that are associated with the biological functions of the tissue. Interestingly, in mammals CGI methylation levels were found to be more similar in promoter regions between tissue types compared to intra and intergenic CGIs [20]. Furthermore, CGIs showing differences in methylation patterns between tissues were overrepresented at loci essential for development (ectoderm and mesoderm development, neurogenesis and segment specification) in human and mouse [20, 21], suggesting a distinct role for CGI methylation in development.

In addition to CpG-methylation, CHG and CHH trinucleotide sites (where H represents any nucleotide but G) can be methylated as well, and this type of methylation is reported for somatic tissues including brain, muscle and placenta [6, 22–24]. Non-CpG methylation accumulates during early postnatal development in the mammalian brain and seems to be driven specifically by the DNA methyltransferase *DNMT3A* [25]. CpA dinucleotide sites are predominantly methylated compared to CpT and CpC sites in mammals, and are starting to be linked to gene regulation in the brain [25, 26]. In addition, the methyl CpG binding protein 2 (MeCP2) binds to methylated non-CpG sites, with a high affinity for mCpA dinucleotide sites. The function of non-CpG methylation and the binding of MeCP2 in brain tissue remains largely unclear, but some studies hypothesize a role for MeCP2 as a transcriptional repressor [27], whereas others propose a role as a transcriptional activator [28].

To expand our understanding of DNA methylation and its possible functions in birds, we investigated both CpG and non-CpG whole genome DNA methylation in brain and blood of a passerine bird (great tit, *Parus major*). We specifically assess methylation patterns and their functional roles in relation to gene features, CGIs, and TEs.

## Results

### The great tit methylome

**Fig. 1:**
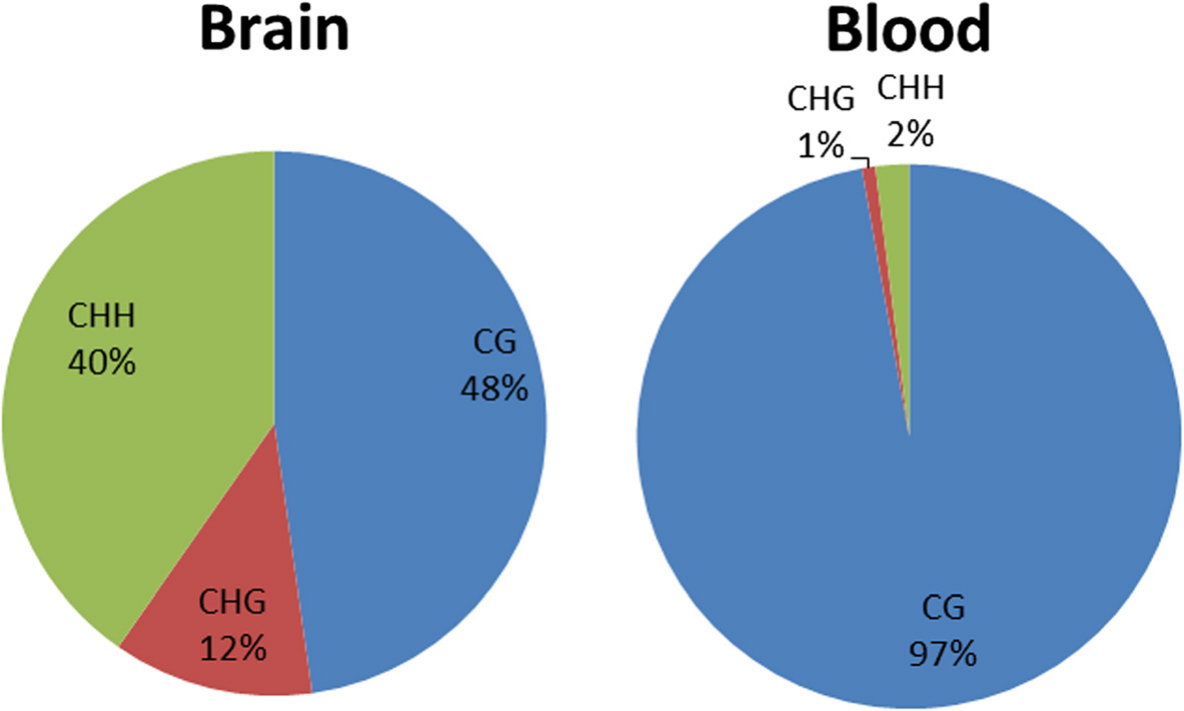
Relative proportion of methylated Cs (methylation level >10 %) in brain and blood for three sequence contexts. The majority of methylated Cs were located at CpG sites in blood, compared to CHH and CHG sites in brain

**Fig. 2:**
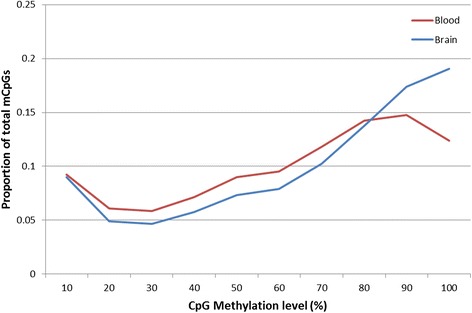
Distribution of methylation level in blood and brain for mCpG-sites. Methylated CpG sites (methylation level > 0 %) covered in both tissues are divided into ten bins according to their methylation level on the x-axis. The y-axis indicates the proportion of total CpGs that are methylated within each bin. A higher abundance of hypermethylated (methylation level > 80 %) CpG sites was observed in brain compared to blood

**Fig. 3:**
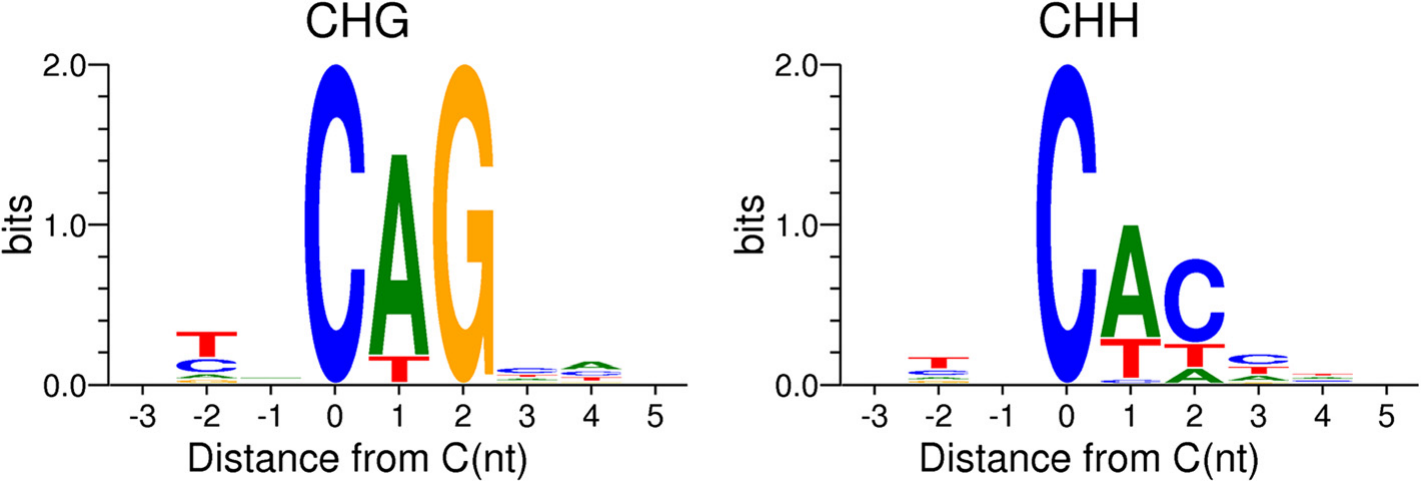
CHG and CHH methylation sequence motifs. Occurrence of nucleotides are given (bits) relative to the distance from the C nucleotide. CpA sites are predominantly methylated (methylation level >10 %) at both CHG and CHH sites, whereas methylated CpC sites are very rare. Cytosine is the most dominant third nucleotide at CHH methylated sites

We performed whole genome bisulfite sequencing in brain and blood samples from a single adult male great tit recently used for genome assembly and annotation [6]. A total of 12.2 (blood) and 10.6 (brain) million CpG sites with a minimum depth of 10x were covered, representing 80 and 69 % of the total CpG sites in the genome, respectively. We observe a higher average CpG methylation level (the ratio of methylated reads to all reads covering a specific site) in brain compared to blood, based on 10,246,241 CpG sites covered (>10x) in both tissues (50.0 and 42.7 %, respectively; Table 1). The large majority of covered CpG-sites were methylated (relative methylation >10 %) in both brain (73.0 %) and blood (70.2 %). These numbers are consistent with previous findings in mammals [29, 30]. We observe significant non-CpG methylation in brain (CHG: 3.4 %, CHH: 5.5 %) but not in blood (Table 1). For blood, 97 % of methylated Cs were derived from CpG sites (Fig. 1). In brain, the majority of methylated Cs (52 %) were located at non-CpG sites, but with generally lower methylation levels. The higher general methylation level in brain is due to a larger proportion of fully methylated CpG-sites (>80 %) (Fig. 2). Similar analysis for non-CpG methylation in brain shows that both CHG and CHH sites have generally no or very low methylation levels (<20 %) and similar distributions (Additional file 1: Figure S1). The majority of non-CpG methylation in the great tit brain occurs at CpA dinucleotide sites (75 %), representing 72 and 88 % of methylated CHH and CHG sites, respectively (Table 2, Additional file 1: Figure S2). Additional non-CpG methylation mainly occurs at CpT sites (22 %), with CpC dinucleotide sites being rarely methylated (3 %). Furthermore, the average methylation level for CpA sites is 3.4 %, 1.16 % for CpT sites, and only 0.31 % for CpC sites (Table 2). Further sequence analysis revealed a dominant CAC sequence motif for CHH methylated sites (Fig. 3). These motifs are consistent with previous findings in mammals [26, 31], but have not been described previously in Aves.

**Table 1:** Methylation profiles in blood and brain. Methylation density describing the proportion of methylated sites (>10 %) in the genome

Site	Sample	Covered sites	Average methylation level for shared sites (%)	Methylated sites (>10 %)	Methylation density (%)
CpG	Blood	12208277	42.74	8567823	70.18
	Brain	10565030	50.01	7714048	73.01
CHG	Blood	72303421	0.09	69783	0.10
	Brain	56072687	1.35	1926491	3.44
CHH	Blood	171057234	0.09	175551	0.10
	Brain	118239257	1.94	6506253	5.50

**Table 2:** Non-CpG dinucleotide methylation level. Table shows dominant non-CpG methylation at CpA sites in both CHG and CHH trinucleotide sites

CpH	Average methylation level (%)	# Methylated CHG sites	# Methylated CHH sites
CpA	3.4	1691994	4655570
CpT	1.16	225529	1645482
CpC	0.31	8968	205201
Total	1.75	1926491	6506253

**Fig. 4:**
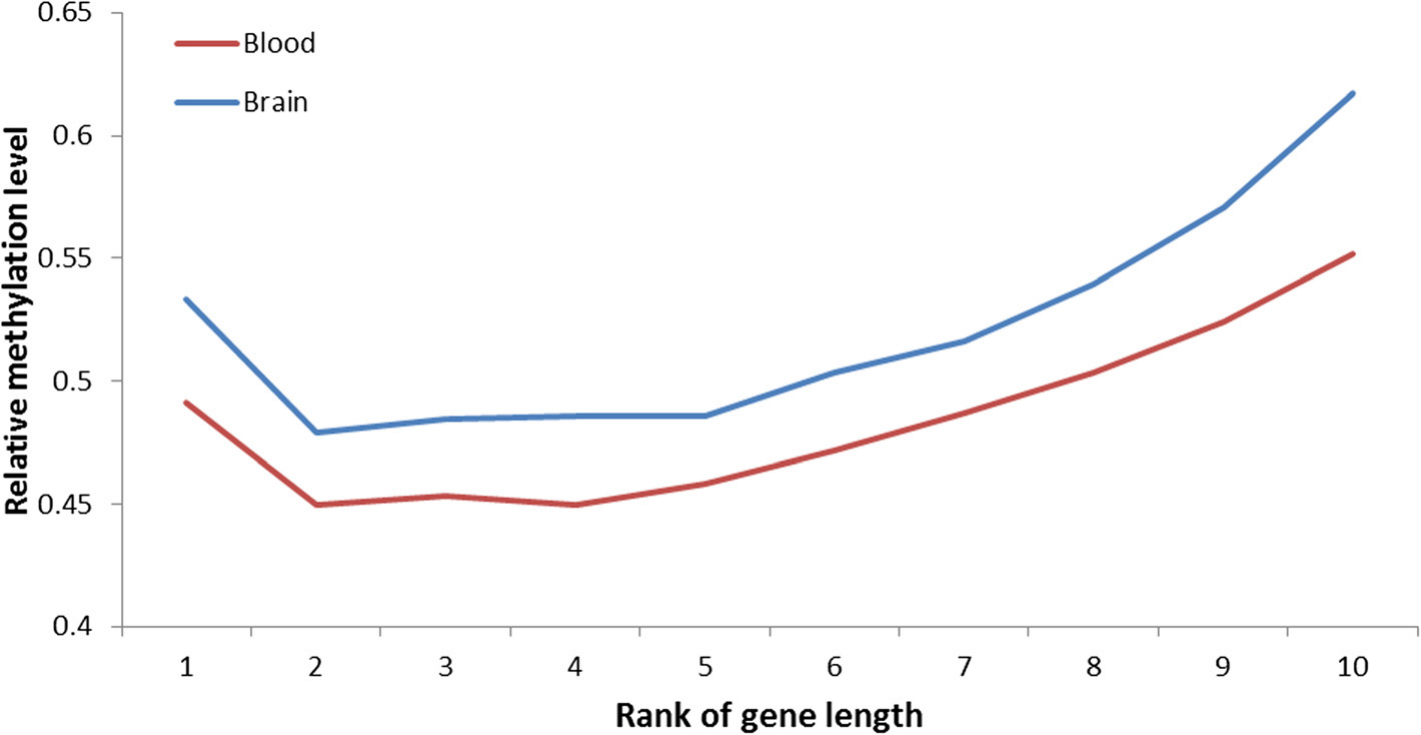
Relationship between CpG methylation level and gene length. Genes were divided into ten bins according to their length. The figure shows a positive trend between average methylation level and gene length

In order to assess the methylation levels in genic regions we divided the genes into specific gene features: TSS (300 bp upstream - 50 bp downstream of the annotated TSS), five prime untranslated region (5’UTR), GB, coding sequence (CDS), introns, three prime untranslated regions (3’UTR) and transcription termination site (TTS; 50 bp upstream - 200 bp downstream of the annotated TTS). Methylation levels were further calculated in the context of gene regions by dividing each gene into overlapping sliding windows and including the 10 kb upstream and downstream regions (Additional file 1: Figures S3, S4). We found low methylation levels in TSS and 5’UTR regions and higher methylation levels in the GB increasing towards the 3’UTR before decreasing again near the TTS. We found higher methylation levels in coding regions compared to intronic regions (Additional file 1: Figure S5) and longer genes have generally higher CpG methylation levels (Fig. 4).

### Differential CpG methylation between blood and brain

We identified genes differentially methylated between brain and blood in three gene features; TSS, GB, and TTS. Based on the fold change calculated from the relative methylation in the two tissue types, we extracted the 5 % of genes with most extreme methylation fold changes (upper and lower 2.5 %) between tissues for enrichment analysis (Table 3). For TSS, GB, and TTS these sets included 382, 410, and 289 genes, respectively. We found very little overlap in the set of differentially methylated genes from the three gene features (Additional file 1: Figure S6). Gene ontology (GO) enrichment analysis showed that genes with a hypomethylated TSS in the brain compared to blood were enriched in categories linked to regulation of axonogenesis, neuronal synaptic plasticity, neurogenesis, and spindle localization (Additional file 2: Table S1). In contrast, genes with a hypomethylated TSS in blood compared to brain were largely enriched in categories linked to lymphocyte and leukocyte cell activation, immune response, and hemopoiesis (Additional file 2: Table S2). Many of these enriched biological processes correspond directly to the specific functions of the examined tissues. Moreover, this result suggests that, at least in brain and blood, genes are generally TSS-hypomethylated in the tissue they are expected to be expressed in. Genes with hypermethylated GBs in brain compared to blood are involved in the (positive) regulation of transcription, development (cell fate commitment, regionalization, embryonic and organ development/morphogenesis), and regulation of immune system process (Additional file 2: Table S3). GB hypomethylated genes in brain compared to blood are involved in transcription, RNA-splicing, and translation (Additional file 2: Table S4). These results suggests that genes involved in transcription, splicing, and translation are generally GB-hypomethylated in brain to enhance their transcription, while genes important for developmental processes are GB-hypermethylated to silence their transcription, supported by significant differences in brain gene expression data between the two sets (TSS; *p* < 1x10^−10^, GB; *p* < 1x10^−9^, TTS; *p* < 0.01; Table 3). Genes with hypermethylated TTS in brain compared to blood are involved in the negative regulation of transcription, opposite of GB-hypermethylated genes (Additional file 2: Table S5). The difference in expression in relation to brain methylation supports the observed negative correlation between methylation levels and gene expression in all gene features, earlier described by Laine et al. 2016 [6] (Additional file 1: Figure S7).

**Table 3:** Differentially methylated gene features between blood and brain

Feature	Covered	2.5 %	Brain hypomethylated FPKM	Brain hypermethylated FPKM
TSS	15284	382	50.8/25.15	17.7/4.5
GB	16413	410	90.1/35.8	15.9/2.3
TTS	11573	289	67.7/22.1	30.9/9.3

We used the upper and lower 2.5 % of genes based on fold-change to identify differentially methylated genes with the sum of both average methylation levels > 10 % in order to exclude hypomethylated genes in both tissue types. The fourth (brain hypomethylated) and fifth (brain hypermethylated) column show average and median brain gene expression values expressed as fragments per kilobase of transcript per million mapped fragments (FPKM) for the set of genes with differentially methylated gene features

### CGI CpG methylation

We divided the CGIs into three groups according to the position of the CGI: promoter associated CGIs (2 kb upstream - 500 bp downstream of annotated TSS), intragenic CGIs, and intergenic CGIs. While promoter CGIs in general tend to be hypomethylated, intragenic CGIs tend to have higher methylation in both tissues (Additional file 1: Figure S8). Moreover, when comparing the methylation levels of these CGIs in blood and brain, we found fewer differentially methylated CGIs in promoter regions (11.9 %) compared to intragenic and intergenic CGIs (23.0 and 25.3 %, respectively; Table 4). We found that genes associated with hypermethylated CGIs (both promoter and intragenic) in brain compared to blood are largely involved in developmental processes (morphogenesis, cell differentiation, organ development, embryonic development, and system development; Additional file 3: Tables S1, S2). These results are consistent with previous studies where differential methylation across tissues was found in genes linked to development [20, 21]. Genes associated with hypomethylated promoter CGIs in brain compared to blood are involved in the protein secretion of platelet, neurotrophin production, and regulation of protein targeting to membrane (Additional file 3: Table S3), while genes with hypomethylated intragenic CGIs in brain are enriched for categories linked to (neuron) development and differentiation (cell differentiation, system development, generation of neurons and neurogenesis; Additional file 3: Table S4). We observe significantly higher brain expression of genes associated with both promoter and intragenic hypomethylated CGIs compared to hypermethylated CGIs in brain (Promoter: *p* < 1x10^−05^, Intra: *p* < 1x10^−07^; Additional file 1: Table S1).

**Table 4:** Differentially methylated CGIs in promoter, intragenic and intergenic regions

CGIs	#	%	Differentially methylated	%	Brain hypermethylated	Brain hypomethylated	Associated genes
Total	33054	100.0	6877	20.8	4622	2255	3652
Promoter	9985	30.2	1186	11.9	748	438	1357
Intragenic	8954	27.1	2055	23.0	1246	809	2295
Intergenic	14820	44.8	3743	25.3	2677	1066	0

We calculated the relative methylation levels for CGIs with at least three CpG sites, and determined differentially methylated CGIs using Fisher’s exact test. Table shows the number of differentially methylated CGIs and associated genes in blood and brain

### Non-CpG gene methylation in brain tissue

To discover if there is a functional signal related to non-CpG methylation we divided all genes according to their non-CpG methylation level in brain and used the upper and lower 2.5 % to perform GO enrichment analysis. Genes with no or very low non-CpG methylation (lower 2.5 %) are largely involved in transcriptional/translational processes (gene expression, mRNA metabolic process, ribosome biogenesis, and translation; Additional file 4: Tables S1-S3). The genes with the highest methylation (upper 2.5 %) in terms of TSS and TTS non-CpG methylation are mainly involved in system development, glial cell differentiation, gliogenesis and glial cell fate specification (Additional file 4: Tables S4, S5), while genes with highly non-CpG methylated GB are frequently involved in processes linked to the immune system (adaptive immune response, leukocyte mediated immunity, B cell mediated immunity) and cell adhesion (Additional file 4: Table S6). Genes in the lower 2.5 % have significantly higher expression (TSS; *p* < 0.001, GB; *p* < 1x10^−11^, TTS; *p* < 1x10^−06^) compared to the highly non-CpG methylated genes (Additional file 1: Table S2). In addition, we found a positive correlation between non-CpG methylation level and gene length (Additional file 1: Figures S9, S10), a pattern consistent with recent reports in mammals [32, 33]. This suggests that the observed differences are biased towards longer genes, where genes involved in protein, DNA, and RNA metabolism are generally shorter compared to genes in the upper non-CpG methylation percentiles (involved in gliogenesis, cell-adhesion or immune response). Additionally, a negative correlation between non-CpG methylation levels and gene expression was observed in all gene features, earlier described by Laine et al. 2016 [6] (Additional file 1: Figure S11).

### Transposable element methylation

**Fig. 5:**
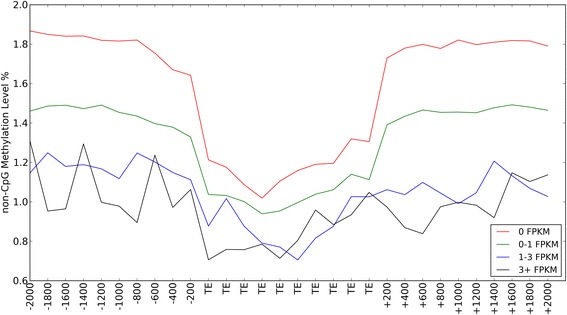
Non-CpG methylation in relation to TE expression in the brain. Figure shows the non-CpG methylation level in TEs, 2 kb upstream, and 2 kb downstream regions in relation to expression, presented as fragments per kilobase of transcript per million mapped fragments (FPKM). Active TEs (FPKM > 0) show lower non-CpG methylation levels compared to inactive TEs (FPKM = 0)

We assessed DNA methylation in 26,834 TEs (mainly LINE/CR1 and LTR/ERVL elements) divided into short interspersed elements (SINE), long interspersed elements (LINE), and LTR retrotransposons. CpG sites within TEs are largely hypermethylated in both brain and blood (Additional file 1: Figure S12). The CpG methylation levels in TEs and upstream and downstream 2 kb flanking regions in brain are significantly higher than in blood, with the difference in average methylation reaching up to 20 %. Interestingly, the opposite pattern was observed for non-CpG methylation, with decreased non-CpG methylation in TEs compared to their flanking regions in brain (Additional file 1: Figure S13). Additionally, TE expression shows a negative correlation with non-CpG methylation level (Spearman’s rho < −0.11, *P* < 1x10^−15^; Fig. 5). This negative correlation is even stronger in the 2 kb upstream (Spearman’s rho < −0.20, *P* < 1x10^−15^) and downstream regions (Spearman’s rho < −0.19, P < 1x10^−15^). Surprisingly this negative correlation was not observed for CpG methylation (Spearman’s rho: TE-body = 0.033, upstream = −0.0056, downstream = −0.0023; Additional file 1: Figure S14). The methylation patterns did not differ between the different types of TEs.

### Independent validation of RNA-seq and WGBS results

In order to validate the RNA-seq gene expression results, we performed qPCR on 11 randomly chosen genes using RNA isolated from whole brain tissue of the same individual, as well as five additional individuals. The results revealed a highly significant positive correlation between the RNA-seq and qPCR gene expression results (Pearson correlation; *r* = 0.753, *p* < 0.001)(Additional file 1: Figure S15). In order to validate the WGBS methylation results, we generated reduced representation bisulfite sequencing (RRBS) data using DNA isolated from blood samples from two additional individuals. An average non-CpG methylation level of <0.4 % was observed, with only ~2 % of the methylated sites (relative methylation >10 %) derive from non-CpG sites in the two RRBS samples (Additional file 1: Table S4), confirming the lack of non-CG methylation in the blood. Furthermore, we used the two RRBS samples to perform correlation analysis on 2000 randomly selected CpG sites (coverage > 10x in all samples) ranging in methylation level between 0 and 100 % in the WGBS sample. We observed a high correlation between the WGBS and both RRBS samples, with Pearson correlation coefficients of 0.758 (*p* < 0.001) and 0.767 (*p* < 0.001), respectively (Additional file 1: Figure S16). Together, these results validate the RNA-seq and WGBS results presented here.

## Discussion

Until recently, chickens were the only birds with DNA methylation information available [34]. The recent publication of the great tit genome has revealed methylation patterns analogous to those of mammals [6]. However, the observed dominant sequence motifs for non-CpG methylation have not been previously described in the Aves class. We have shown differential methylation levels between brain and blood for groups of genes linked to the functions of those tissues. Furthermore, differentially methylated CGIs between brain and blood that are located in promoter and intragenic regions are overrepresented in genetic loci essential for development. TEs are hypermethylated at CpG sites but hypomethylated at non-CpG sites. Interestingly, TE expression in brain tissue negatively correlates with non-CpG methylation, but not with CpG methylation. This novel finding provides new insights in the mechanism of TE silencing by methylation in the great tit brain.

We observed a positive correlation between gene length and methylation level (both CpG and non-CpG). One explanation is that larger genes can compensate for lowly methylated TSS and 5’UTR regions, since they consist of a relatively larger GB. This, however, does not explain that genes in the first size rank (smallest genes) show higher methylation in CpG context. Since single exon genes will be more predominant in the lower rank, with the methylation levels being higher in coding-sequences compared to intronic regions, this might explain the unusually high methylation levels of the smallest gene set.

There is a very strong association between TSS hypomethylation and gene function; genes with brain related functions were also hypomethylated in brain TSS, supporting that decreased TSS methylation is involved in very specific gene regulation. Interestingly, this signal was not observed for promoter CGIs. In general, methylation levels are conserved in promoter CGIs between the two tissues, being hypomethylated in both brain and blood, supporting the generalization that CGI methylation silences promoters. Intragenic CGIs tend to show higher methylation levels. Hypermethylated promoter and intragenic CGIs in brain are associated with genes involved in development. One hypothesis is that CGI hypermethylation in genomic loci linked to development could silence transcription after brain development. Following human germ cell development, the genome is demethylated and remethylated [35]. The opposing hypermethylation of intragenic CGIs in adult brain and hypomethylation in blood could further be explained by the negative correlation between gene body methylation and expression in brain [10] and the positive correlation in blood [36]. However this does not explain the difference between adult blood and brain methylation levels at promoter CGIs.

Non-CpG methylation in (passerine) birds seems to be restricted to certain tissue types and we only found this in brain. No or very low non-CpG methylation was found for genes involved in transcriptional and translational processes. In addition, these genes show higher expression compared to the average. We therefore suggest that highly expressed genes have very little or no non-CpG methylation to enhance transcription. We found a dominant CAG and CAC sequence motif for both CHG and CHH methylated sites, as previously described in mammals [26, 31]. This supports the existence of a shared non-CpG regulatory mechanism between birds and mammals. We could even argue that non-CpG methylation occurs predominantly on CWG and CWH sites (W = A or T), because CC dinucleotide sites are hardly methylated. The observed dominant motifs are associated with a stronger binding affinity of MeCP2 (especially the methylated CpA dinucleotide sites) in mammals [32]. MeCP2 has also been shown to preferentially silence longer genes enriched with methylated CpA sites [32]. This could explain why we find no or very low CpA methylation in genes involved in protein, DNA, and RNA metabolism (which are generally shorter genes), but do find it in longer genes (e.g. genes involved in cell adhesion). However, the exact function of MeCP2 binding to non-CpG methylated sites remains largely unclear and is associated with both transcriptional silencing and activation [27, 28].

The large differences we found in general methylation levels between brain and blood can be explained by a larger proportion of fully methylated CpG sites in brain, where in blood moderate methylation is more abundant. This dissimilarity might in part be attributed to hypermethylated TEs in brain, which are methylated at lower levels in blood. Methylation of TEs decreases or inhibits transpositional activity. Previous studies observed large differences in TE methylation levels between tissues [37], although not specifically for blood and brain. Opposite to CpG methylation, we found decreased non-CpG methylation in TEs compared to their flanking regions. In addition, our observation that non-CpG methylation negatively correlates with TE activity suggests a distinct role for non-CpG methylation in TE silencing in the brain. Surprisingly, we did not observe any correlation between CpG methylation and TE activity. Recent studies have shown that TEs are more active in brain tissue compared to other tissues in mammals [38]. Based on our results, we hypothesize that non-CpG methylation is involved in, and is perhaps even the main regulator of TE silencing in brain.

It is important to note that although we observe a role for brain methylation in regulating gene expression and TE activity, the brain consists of several highly distinctive regions with a variety of functions. Therefore, the results presented here must be taken as the average for whole brain, and cannot be directly correlated with individual brain regions. Moreover, since we only used two tissue types in our analysis, we can only draw conclusions about differences among these tissues. To make more general conclusions about tissue-specificity, more tissues need to be included in the future. Nevertheless, these results provide new insights and present new possibilities to further exploration of the role of methylation (especially the role of non-CpG methylation in TEs) in specific brain regions. In addition, while whole genome bisulfite sequencing is considered the gold standard technique for analyzing genome-wide DNA methylation patterns, the high cost associated with producing such datasets limits the number of biological replicates in studies such as this one. Therefore, although performing statistical analysis at the genic level allows for robust statistical analysis within a single sample, and no non-CpG methylation was detected in the whole blood sample (as expected), further studies in additional individuals are required to confirm the results presented here.

## Conclusion

We show distinct methylation patterns of two tissue types in the great tit. In addition, we observe a clear link between methylation and the regulation of gene expression and TE activity in the brain. The dominant CAG and CAC sequence motifs for brain non-CpG methylation, which are also observed in mammals, suggests a conserved non-CpG regulatory mechanism is present between birds and mammals. From our results, we hypothesize a distinct function for CpG and non-CpG methylation in TE silencing in the brain. This theory is supported by the contrast between CpG and non-CpG methylation in TEs. Moreover, a negative correlation between non-CpG methylation and TE activity (not found for CpG methylation) shows that non-CpG methylation is involved in TE silencing and perhaps the dominant regulatory form of methylation in the brain.

## Methods

### Sample collection

Whole brain was collected from a single adult male bird, snap-frozen, and stored in RNAlater (Thermo Fisher Scientific) until processing. This bird is the same as the reference genome individual [6]. Whole blood was collected from the same individual from the carotid artery after euthanization. Approval was received for this from the Animal Experiment Committee from the Royal Netherlands Academy of Sciences (DEC-KNAW) under protocol number CTE-0705 Adendum I.

### Whole genome bisulfite sequencing library preparation and sequencing

DNA was extracted from whole blood using a Gentra Puregene Kit (Qiagen, USA) following the manufacturer’s protocol. Homogenized whole brain was incubated overnight at 55 °C in 750 μl Cell Lysis Solution (Gentra Puregene Kit, Qiagen, USA) with 20 μl proteinase K. 250 μl of this lysed tissue was added to 250 ul Cell Lysis Solution. To remove excess of fat and proteins, 500 μl 24:1 chloroform:isoamylalcohol was added and mixed until homogeneous, followed by 10 min centrifugation at 12.000x g after which the upper layer was collected. Cell Lysis Solution was added to this upper layer until 500 μl of sample liquid was obtained and total DNA was extracted according to the manufacturer’s protocol. DNA was stored in DNA Hydration Solution (Qiagen, USA), and the concentration was determined with a Nanodrop 2000 (ThermoThermo Scientific, USA). DNA was sheared using a Covaris E210 device to ~700 bp peak fragment sizes. One microgram sheared and purified DNA including 1 ng sheared Lambda DNA was used for end repair, adenylating and adapter ligation according to illumina TruSeq LT DNA sample preparation guide. Adapter Ligated DNA was purified using AmpureXP beads (Agencourt) and subsequently used for bisulfite conversion according to EpiTect Plus Bisulfite workflow (Qiagen) with small modifications i.e. three additional rounds of denaturation’s (5 min at 95 °C each) and incubations (60 °C for 25, 85 and 125 min respectively). Converted DNA was purified following manufacturer’s instructions (EpiTect) and split over three parallel reactions for library amplification using Pfu Cx hotstart DNA polymerase (Agilent Technologies) and 18 PCR cycles. PCR products were pooled per sample and final amplified bisulfite converted libraries were quantified using Qubit (Invitrogen) and Bio analyzer DNA 1000 chip (Agilent Technologies). Libraries were used for clustering on two partial flowcells on an Illumina HiSeq 2000 system generating 358.3 M (72.4Gbp) and 292.4 M (59.1Gbp) paired-end reads (101 bp) for brain and blood, respectively.

### Whole genome bisulfite sequencing analysis

Raw reads were trimmed for quality (>20) and adapter sequences using trim_galore v.0.1.4 (http://www.bioinformatics.babraham.ac.uk/projects/trim_galore/), producing a cleaned set of 334.8 M and 274.8 M paired end reads for brain and blood, respectively. These reads were aligned to the great tit reference genome [6] using BS-Seeker v2.0.6 [39] with Bowtie2 v2.1.0 using the local alignment mode [40]. In total, 95,79 and 98.04 % of the genome was covered with an average depth of 31.89x (52 % mapping rate) and 33.04x (64 % mapping rate) in brain and blood, respectively. Methylation levels (defined as the ratio of methylated/total reads at a given site) were determined using the methylation call scripts from BSseeker2. All downstream analysis was performed using sites covered by a minimum of 10 reads.

### Methylation variation across gene features

Each gene is subdivided into TSS (300 bp upstream, 50 bp downstream of annotated TSS), GB (representing the full length of the annotated gene), and TTS (50 bp upstream, 200 bp downstream of annotated TTS). Transcripts with similar TSS and TTS boundaries were merged to use a set of unique transcripts for downstream analysis. We calculated the fold-change difference in average CpG methylation level between blood and brain in all three gene features (TSS, GB, TTS) where the sum of the relative methylation level in blood and brain is at least 10 % (to exclude hypomethylated gene features in both tissues) and with at least three CpG sites covered. We used the upper (hypermethylated) and lower (hypomethylated) 2.5 % of genes to define differentially methylated regions. We calculated the average non-CpG methylation in blood and brain for TSS, GB, and TTS with at least three non-CpG sites covered. We divided each region into 40 groups of percentiles based on their non-CpG methylation levels using a custom python script and the upper (40th) and lower (1st) percentiles were defined as differentially methylated. The GB was further subdivided into 4 different GB features using custom python scripts; 5’UTR, CDS, intronic sequences, and 3’UTR. We calculated the average methylation levels for both CpG and non-CpG sites covering these GB features. In addition, we used a sliding window approach to correlate the methylation level with gene expression in brain (Additional file 1: Figures S7-S11). We used three different gene regions; the GB, 10 kb upstream, and 10 kb downstream of the gene boundaries, For each region, we used 40 overlapping sliding windows (5 % of the regions length) with an overlap of 2.50 %. We subdivided the genes in 10 percentiles based on gene length to assess the correlation between gene length and relative methylation level (over the full length of the annotated gene).

### Non-CpG methylation sequence motifs

Custom python scripts were used to retrieve sequence motifs (3 bp upstream and 5 bp downstream of the methylated site) and WebLogo3.3 was used to build sequence logos [41].

### CGI prediction and differential methylation

CGIs were predicted using the cpgplot software, part of the EMBOSS package (version 6.6.0.0) applying default settings (−window 100 -minlen 200 -minoe 0.6 -minpc 50). In total 33,131 CGIs were identified in the genome. CGIs were associated with a promoter if the CGI was annotated within 2 kb upstream to 500 bp downstream of a gene’s annotated TSS. A CGI was considered intragenic if annotated within the boundaries of a gene, or if it completely covered the gene. We calculated the relative methylation levels for CGIs with at least three CpG sites, and differentially methylated CGIs were determined using a fisher exact test. Bonferroni correction was used to account for multiple testing, q-value < = 0.05 is considered significant.

### RNA-seq library preparation and sequencing

RNA was extracted from homogenized whole brain using the miRNeasy mini kit (Qiagen) following the manufacturer’s protocol. For library preparation we used the TruSeq Stranded RNA Sample Preparation Kit (Illumina) using a barcoded adapter. Sequencing was performed on an Illumina HiSeq 2000. This resulted in 229.6 M (46.4Gbp) paired-end 100 bp RNA-seq reads.

### RNA-seq analysis

FastQC (http://www.bioinformatics.babraham.ac.uk/projects/fastqc/) was used to check the quality of the sequences and low-quality bases were trimmed off using Fastq-mcf [42]. In total, 200.8 M trimmed paired end reads were aligned (84.7 % overall mapping rate) against the reference genome with Tophat v2.0.10 using –max-multihits 20, --read-realign-edit-dist 0,--mate-inner-dist 50,--mate-std-dev 150 [43]. Tophat analysis included a pre-alignment to the reference genome to filter out reads extending the maximum number of alignments (-M option) followed by alignment to the reference transcriptome (-G) and alignment to the genome. Transcript assembly and quantification was done with Cufflinks v2.2.0 including the annotation (-g option),--overlap-radius 5 and --intron-overhang-tolerance 5. Expression values were extracted from the cufflinks output and represented as fragments per kilobase of exons per million mapped reads (FPKM) [43]. We used a two sample *t*-test assuming equal variances to test for significant gene expression differences between differentially methylated gene sets. Spearman’s correlations between gene expression and methylation were calculated using R (version: 3.0.2).

### Gene ontology (GO) analysis

We performed blastp (e-value 0.1) [44] to human, mouse, and chicken, and InterProScan [45] on the total great tit gene set. Out of 16,424 transcripts, 15,186 were assigned to a total of 247,144 GO-terms (Additional file 1: Table S3). These results were imported into Blast2GO to assign GO-terms for each individual gene [46]. We calculated the fold-change difference in average CpG methylation level between blood and brain in all three gene features (TSS, GB, TTS) where the sum of the relative methylation level in blood and brain is at least 10 % (to exclude hypomethylated gene features in both tissues) and with at least three CpG sites covered. We used the upper (hypermethylated) and lower (hypomethylated) 2.5 % of gene features to define differentially methylated regions for gene ontology analysis. We calculated the average non-CpG methylation in blood and brain for TSS, GB, and TTS with at least three non-CpG sites covered. We divided the regions into 40 groups of percentiles based on their non-CpG methylation levels (minimum 3 non-CpG sites) using a custom python script and the upper (40th) and lower (1st) percentiles were defined as differentially methylated and used for GO-enrichment analysis. The CytoScape plugin BINGO [47] was used for GO enrichment analysis using a hypergeometric test to calculate p-values and a Benjamin & Hochberg False Discovery Rate (FDR) correction to calculate q-values [47]. The GO annotations from Blast2GO were used as the reference data set. GO-terms with a q-value less than or equal to 0.05 were considered enriched.

### Transposable elements

Transposable elements (TE) were annotated using RepeatMasker (v4.0.3) [48] with RepBase (update: 20130422) [49]. In total, 26,834 TEs covering 2.1 % (21.3 Mb) of the genome were used in the analysis with TEs smaller than 500 bp excluded. We used a sliding window approach to assess the relative methylation levels of TEs by divided them into three different regions; the TE, 2 kb upstream and 2 kb downstream. Each region was subdivided into ten overlapping sliding windows (20 % of the regions length) with an overlap of 10 %, and average methylation levels were calculated for each sliding window. We used Tophat version 2.0.10 to uniquely align the RNA-seq reads (84.3 % overall mapping rate) to the reference genome [43] with following settings: max-multihits 1,--read-realign-edit-dist 0,--mate-inner-dist 50 and --mate-std-dev 150. TE expression was assessed using the cufflinks --GTF option with a GFF containing all identified TEs [43] and --overlap-radius 5,--intron-overhang-tolerance 5. TE expression values (FPKM) were extracted from the Cufflinks output. Spearman’s correlations between TE expression and methylation were calculated using R (version: 3.0.2).

### Validation of RNA-seq via quantitative real-time PCR (qPCR)

RNA was extracted from hypothalamus tissue of five great tits collected for another study (Verhagen unpublished.). Punches were taken from the hypothalamus region using the method of Perfito et al. 2012 [50]. In short, brain tissue was cut on a cryostat. 3 mm circular punches were sampled from the hypothalamus region, resulting in sampling equal volumes from each individual brain. Brain punches were immediately added to 1 ml TRIzol Reagent (Thermo Fisher Scientific, Waltham, USA), homogenized and stored at -80C until extraction. Total RNA of brain punches was extracted using the method of TRIzol Reagent. For these five individuals and the single reference individual used for the other analyses (for RNA extraction see RNA-seq library preparation and sequencing) 354 ng of RNA was used to synthesize first-strand cDNA with QuantiTect Reverse Transcription Kit (Qiagen, Venlo, The Netherlands) following the standard protocols. RNA concentration was quantified using a Nanodrop 1000 spectrophotometer (Thermo Fisher Scientific) and an AATI Fragment Analyser (Advanced Analytical Technologies, Heidelberg, Germany). The expression level of 12 genes was further investigated by qPCR. The qPCR analysis was performed with PowerUp SYBR Green Master Mix (Thermo Fisher Scientific) in a C1000 Touch CFX96 (Biorad, Veenendaal, the Netherlands) Primers used for qPCR analysis are given in Additional file 1: Table S5. Great tit GAPDH (augustus_masked-chr1-processed-gene-22.8) was used as a standard control. The qPCR program was performed as follows: 50 °C (2 min), 95 °C (2 min), (95 °C (15 s), 60 °C (1 min))x40 cycles. PCR fragment sizes for the 12 genes were visualized on 1 % agarose gel to verify their band size and specificity.

### Validation of WGBS via RRBS

For this we used blood samplings of two great tits originating from the F2 inter-cross between lines selected for high and low levels of exploratory behaviour [51]. Blood samples of 10 ul were collected when the birds were 40 days old and stored in 1 ml Cell Lysis Solution (Gentra Puregene Kit, Qiagen, USA). Total DNA was prepared by using 250 μl of the stored blood samples with 750 μl Cell Lysis Solution (Gentra Puregene Kit, Qiagen, USA) incubated with proteinase K at 55C overnight, followed by DNA extraction following the manufacturer’s protocol. DNA was stored in DNA Hydration Solution (Qiagen, USA). Integrity of the DNA as well as absence of RNA was verified by running and visually analyzing 1.5 ul of DNA on a 1 % agarose gel next to a DNA ladder. High-quality genomic DNA (1 μg) was sent to the Carver High-Throughput DNA Sequencing and Genotyping Unit (University of Illinois, Urbana, IL, USA) for generation of RRBS libraries following standard protocols. Briefly, DNA was restriction digested using the methyl-insensitive restriction enzyme Mspl, and the resulting fragments were size-selected (20–200 bp) using agarose gel electrophoresis. Size-selected DNA was bisulfite-treated with the EpiTech Bisulfite Kit (Qiagen, Valencia, CA, USA) and column-purified. The final libraries were quantified using Qubit (Life Technologies, Carlsbad, CA, USA) and the average size was determined on an Agilent bioanalyzer DNA7500 DNA chip (Agilent Technologies, Wilmington, DE, USA) and diluted to 10 nM. The 10 nM dilution was further quantitated by qPCR on an ABI 1900 to ensure high accuracy quantification for consistent pooling of barcoded libraries and maximization of the number of clusters in the Illumina flowcell. RRBS Illumina sequencing was performed on libraries multiplexed and loaded onto 8-lane flowcells for cluster formation and sequenced on an Illumina HiSeq2500. The libraries were sequenced to a total read length of 100 bp from one end (single-end sequencing) of the molecules. Raw reads were trimmed using trim_galore v 0.4.1 and aligned to the great tit reference genome [6] using BS-Seeker v2.0.10 in --rrbs mode [39] with Bowtie2 v2.2.7 using the local alignment mode [40]. This resulted in a mappability of 75.43 and 75.94 %. Methylation levels were determined using the methylation call scripts from BSseeker2 with a minimum depth of 10x. 2000 randomly selected CpG sites (coverage > 10x in all samples) ranging in methylation level between 0 and 100 % in the WGBS sample were extracted from the RRBS samples. Pearson correlations on the 2000 randomly selected CpG sites were calculated using R (version: 3.0.2).

## Availability of data and materials

The raw methylome data has been deposited into the NCBI Short Read Archive (SRA) under BioProject PRJNA208335 in the study SRP055861 and are accessible via the following URLs: brain: http://www.ncbi.nlm.nih.gov/sra/?term=SRS964344 blood: http://www.ncbi.nlm.nih.gov/sra/?term=SRS964345. The raw RRBS data are accessible via the following URLs: http://www.ncbi.nlm.nih.gov/sra/?term=SRS1341021 and http://www.ncbi.nlm.nih.gov/sra/?term=SRS1340777. The raw brain RNA-seq data is accessible via the URL: http://www.ncbi.nlm.nih.gov/sra/?term=SRS866013. The version of the genome used in this paper can be found at GenBank under the accession GenBank:JRXK01000000. Datasets supporting the results of this article are also included in the additional files.

## Additional files

Additional file 1: Figure S1. Methylation level distribution for non-CpG sites (>0 %). **Figure S2.** non-CpG dinucleotide methylation preferences. **Figure S3.** CpG methylation level distribution in genes. **Figure S4.** non-CpG methylation level distribution in genes. **Figure S5.** Average CpG methylation in different gene partitions. **Figure S6.** The overlap for brain differentially hypo-methylated (A) and hyper-methylated (B) gene features. **Figure S7.** CpG methylation in relation to gene expression in brain. **Figure S8.** Relative CpG methylation for CGIs divided over three genomic regional classes. **Figure S9.** Relationship between non-CpG methylation level and gene length. **Figure S10.** Average gene length for 40 groups of percentiles of non-CpG methylated genes. **Figure S11.** Non-CpG methylation in relation to gene expression in brain. **Figure S12.** CpG methylation level distribution in TEs and their 2 kb flanking regions. **Figure S13.** Non-CpG methylation level distribution in TEs and their 2 kb flanking region. **Figure S14.** CpG methylation in relation to TE expression in the brain. **Figure S15.** Standardized gene expression from qPCR (Fold Change) as a function of the gene expression calculated from RNA-seq. **Figure S16.** Whole genome bisulfite sequencing (WGBS) vs. Reduced representation bisulfite sequencing (RRBS) in blood. **Table S1.** Average and median gene expression in brain for genes associated with differentially methylated CGIs. **Table S2.** Average and median gene expression levels for upper and lower 2.5 % non-CpG methylated genes (brain). **Table S3.** Blast2GO gene ontology annotation. **Table S4.** Methylation profiles in two blood RRBS samples. **Table S5.** Primer information for the genes used for qPCR validation. (DOCX 1636 kb) Additional file 2: Full GO-enrichment analysis for differentially methylated gene features: **Tables S1** to **S6**. (XLSX 169 kb) Additional file 3: Full GO-enrichment analysis for genes associated with differentially methylated CGIs: **Tables S1** to **S4**. (XLSX 130 kb) Additional file 4: Full GO-enrichment analysis for upper and lower non-CpG methylated genes: **Tables S1** to **S6**. (XLSX 126 kb)

## Abbreviations

3’UTR: three prime untranslated region
5’UTR: five prime untranslated region
CDS: coding sequence
CGI: CPG-island
FDR: false discovery rate
FPKM: fragments per kilobase of exons per million mapped reads
GB: gene body
GO: gene ontology
LINE: long interspersed element
qPCR: quantitative real-time PCR
RRBS: reduced representation bisulfite sequencing
SINE: short interspersed element
TE: transposable element
TSS: transcription start site
TTS: transcription termination site
WGBS: whole genome bisulfite sequencing

## References

[1] Auclair G, Weber M (2012). Mechanisms of DNA methylation and demethylation in mammals. Biochimie.

[2] Robertson M, Richards C (2015). Opportunities and challenges of next-generation sequencing applications in ecological epigenetics. Mol Ecol.

[3] Nussey DH, Postma E, Gienapp P, Visser ME (2005). Selection on heritable phenotypic plasticity in a wild bird population. Science.

[4] Pettifor RA, Perrins CM, Mccleery RH (1988). Individual optimization of clutch size in great tits. Nature.

[5] van Oers K, Naguib M (2013). Behavior, physiology, and evolution. Animal personalities.

[6] Laine VN, Gossmann TI, Schachtschneider KM, Garroway CJ, Madsen O, Verhoeven KJ (2016). Evolutionary signals of selection on cognition from the great tit genome and methylome. Nat Commun.

[7] Jones PA (2012). Functions of DNA methylation: islands, start sites, gene bodies and beyond. Nat Rev Genet.

[8] Deaton AM, Bird A (2011). CpG islands and the regulation of transcription. Genes Dev.

[9] Jones PA (1999). The DNA, methylation paradox. Trends Genet.

[10] Wen L, Li XL, Yan LY, Tan YX, Li R, Zhao YY (2014). Whole-genome analysis of 5-hydroxymethylcytosine and 5-methylcytosine at base resolution in the human brain. Genome Biol.

[11] Luco RF, Pan Q, Tominaga K, Blencowe BJ, Pereira-Smith OM, Misteli T (2010). Regulation of alternative splicing by histone modifications. Science.

[12] Chodavarapu RK, Feng SH, Bernatavichute YV, Chen PY, Stroud H, Yu YC (2010). Relationship between nucleosome positioning and DNA methylation. Nature.

[13] Kobayashi H, Sakurai T, Imai M, Takahashi N, Fukuda A, Yayoi O (2012). Contribution of intragenic DNA methylation in mouse gametic DNA methylomes to establish oocyte-specific heritable marks. Plos Genet.

[14] Slotkin RK, Martienssen R (2007). Transposable elements and the epigenetic regulation of the genome. Nat Rev Genet.

[15] Yoder JA, Walsh CP, Bestor TH (1997). Cytosine methylation and the ecology of intragenomic parasites. Trends Genet.

[16] Rabinowicz PD, Palmer LE, May BP, Hemann MT, Lowe SW, McCombie WR (2003). Genes and transposons are differentially methylated in plants, but not in mammals. Genome Res.

[17] Matzke MA, Mosher RA (2014). RNA-directed DNA methylation: an epigenetic pathway of increasing complexity. Nat Rev Genet.

[18] Weber M, Hellmann I, Stadler MB, Ramos L, Paabo S, Rebhan M (2007). Distribution, silencing potential and evolutionary impact of promoter DNA methylation in the human genome. Nat Genet.

[19] Wan J, Oliver VF, Wang GH, Zhu H, Zack DJ, Merbs SL (2015). Characterization of tissue-specific differential DNA methylation suggests distinct modes of positive and negative gene expression regulation. BMC Genomics.

[20] Illingworth R, Kerr A, Desousa D, Jorgensen H, Ellis P, Stalker J (2008). A novel CpG island set identifies tissue-specific methylation at developmental gene loci. PLoS Biol.

[21] Deaton AM, Webb S, Kerr ARW, Illingworth RS, Guy J, Andrews R (2011). Cell type-specific DNA methylation at intragenic CpG islands in the immune system. Genome Res.

[22] Chen PY, Feng SH, Joo JWJ, Jacobsen SE, Pellegrini M (2011). A comparative analysis of DNA methylation across human embryonic stem cell lines. Genome Biol.

[23] Venhoranta H, Li S, Salmon S, Flisikowska T, Andersson M, Switonski M (2014). Non-CpG hypermethylation in placenta of mutation-induced intrauterine growth restricted bovine foetuses. Biochem Bioph Res Co.

[24] Varley KE, Gertz J, Bowling KM, Parker SL, Reddy TE, Pauli-Behn F (2013). Dynamic DNA methylation across diverse human cell lines and tissues. Genome Res.

[25] Lister R, Mukamel EA, Nery JR, Urich M, Puddifoot CA, Johnson ND (2013). Global epigenomic reconfiguration during mammalian brain development. Science.

[26] Shirane K, Toh H, Kobayashi H, Miura F, Chiba H, Ito T (2013). Mouse oocyte methylomes at base resolution reveal genome-wide accumulation of Non-CpG methylation and role of DNA methyltransferases. Plos Genet.

[27] Lyst MJ, Ekiert R, Ebert DH, Merusi C, Nowak J, Selfridge J (2013). Rett syndrome mutations abolish the interaction of MeCP2 with the NCoR/SMRT co-repressor. Nat Neurosci.

[28] Chahrour M, Jung SY, Shaw C, Zhou XB, Wong STC, Qin J (2008). MeCP2, a key contributor to neurological disease, activates and represses transcription. Science.

[29] Ehrlich M, Gama-Sosa MA, Huang LH, Midgett RM, Kuo KC, McCune RA (1982). Amount and distribution of 5-methylcytosine in human DNA from different types of tissues of cells. Nucleic Acids Res.

[30] Lister R, Pelizzola M, Dowen RH, Hawkins RD, Hon G, Tonti-Filippini J (2009). Human DNA methylomes at base resolution show widespread epigenomic differences. Nature.

[31] Guo JU, Su YJ, Shin JH, Shin JH, Li HD, Xie B (2014). Distribution, recognition and regulation of non-CpG methylation in the adult mammalian brain. Nat Neurosci.

[32] Gabel HW, Kinde B, Stroud H, Gilbert CS, Harmin DA, Kastan NR (2015). Disruption of DNA-methylation-dependent long gene repression in Rett syndrome. Nature.

[33] Sugino K, Hempel CM, Okaty BW, Arnson HA, Kato S, Dani VS (2014). Cell-type-specific repression by methyl-CpG-binding protein 2 is biased toward long genes. J. Neurosci.

[34] Li QH, Li N, Hu XX, Li JX, Du Z, Chen L (2011). Genome-wide mapping of DNA methylation in chicken. Plos One.

[35] Mann MR, Bartolomei MS. Epigenetic reprogramming in the mammalian embryo: struggle of the clones. Genome Biol. 2002;3(2):1003–1. 10.1186/gb-2002-3-2-reviews1003 PMC139014 11864375

[36] van Eijk KR, de Jong S, Boks MP, Langeveld T, Colas F, Veldink JH (2012). Genetic analysis of DNA methylation and gene expression levels in whole blood of healthy human subjects. BMC Genomics.

[37] Guo HS, Zhu P, Yan LY, Li R, Hu BQ, Lian Y (2014). The DNA methylation landscape of human early embryos. Nature.

[38] Erwin JA, Marchetto MC, Gage FH (2014). Mobile DNA elements in the generation of diversity and complexity in the brain. Nat Rev Neurosci.

[39] Guo WL, Fiziev P, Yan WH, Cokus S, Sun XG, Zhang MQ (2013). BS-Seeker2: a versatile aligning pipeline for bisulfite sequencing data. BMC Genomics.

[40] Langmead B, Salzberg SL (2012). Fast gapped-read alignment with Bowtie 2. Nat Methods.

[41] Crooks GE, Hon G, Chandonia JM, Brenner SE (2004). WebLogo: a sequence logo generator. Genome Res.

[42] Aronesty E (2013). Comparison of sequencing utility programs. Open Biochem. J.

[43] Trapnell C, Roberts A, Goff L, Pertea G, Kim D, Kelley DR (2012). Differential gene and transcript expression analysis of RNA-seq experiments with TopHat and Cufflinks. Nat Protoc.

[44] Altschul SF, Gish W, Miller W, Myers EW, Lipman DJ (1990). Basic local alignment search tool. J Mol Biol.

[45] Jones P, Binns D, Chang HY, Fraser M, Li WZ, McAnulla C (2014). InterProScan 5: genome-scale protein function classification. Bioinformatics.

[46] Conesa A, Gotz S, Garcia-Gomez JM, Terol J, Talon M, Robles M (2005). Blast2GO: a universal tool for annotation, visualization and analysis in functional genomics research. Bioinformatics.

[47] Maere S, Heymans K, Kuiper M (2005). BiNGO: a cytoscape plugin to assess overrepresentation of gene ontology categories in biological networks. Bioinformatics.

[48] Chen N. Using RepeatMasker to identify repetitive elements in genomic sequences. Current protocols in bioinformatics/editoral board, Andreas D Baxevanis [et al.]. 2004;Chapter 4:Unit 4 10. 10.1002/0471250953.bi0410s05 18428725

[49] Jurka J, Kapitonov VV, Pavlicek A, Klonowski P, Kohany O, Walichiewicz J (2005). Repbase Update, a database of eukaryotic repetitive elements. Cytogenet. Genome Res.

[50] Perfito N, Jeong SY, Silverin B, Calisi RM, Bentley GE, Hau M (2012). Anticipating spring: wild populations of great tits (Parus major) differ in expression of key genes for photoperiodic time measurement. Plos One.

[51] van Oers K, Santure AW, De Cauwer I, van Bers NE, Crooijmans RP, Sheldon BC (2014). Replicated high-density genetic maps of two great tit populations reveal fine-scale genomic departures from sex-equal recombination rates. Heredity.

