# Supplementary material for: Gene and transposable element methylation in great tit (Parus major) brain and blood
Source: BMC Genomics. 2016 May 4;17:332. doi: 10.1186/s12864-016-2653-y (PMC4855439; doi:10.1186/s12864-016-2653-y)
Supplement: Additional file 1: Figure S1. — Methylation level distribution for non-CpG sites (>0 %). Figure S2. non-CpG dinucleotide methylation preferences. Figure S3. CpG methylation level distribution in genes. Figure S4. non-CpG methylation level distribution in genes. Figure S5. Average CpG methylation in different gene partitions. Figure S6. The overlap for brain differentially hypo-methylated (A) and hyper-methylated (B) gene features. Figure S7. CpG methylation in relation to gene expression in brain. Figure S8. Relative CpG methylation for CGIs divided over three genomic regional classes. Figure S9. Relationship between non-CpG methylation level and gene length. Figure S10. Average gene length for 40 groups of percentiles of non-CpG methylated genes. Figure S11. Non-CpG methylation in relation to gene expression in brain. Figure S12. CpG methylation level distribution in TEs and their 2 kb flanking regions. Figure S13. Non-CpG methylation level distribution in TEs and their 2 kb flanking region. Figure S14. CpG methylation in relation to TE expression in the brain. Figure S15. Standardized gene expression from qPCR (Fold Change) as a function of the gene expression calculated from RNA-seq. Figure S16. Whole genome bisulfite sequencing (WGBS) vs. Reduced representation bisulfite sequencing (RRBS) in blood. Table S1. Average and median gene expression in brain for genes associated with differentially methylated CGIs. Table S2. Average and median gene expression levels for upper and lower 2.5 % non-CpG methylated genes (brain). Table S3. Blast2GO gene ontology annotation. Table S4. Methylation profiles in two blood RRBS samples. Table S5. Primer information for the genes used for qPCR validation. (DOCX 1636 kb) [file 12864_2016_2653_MOESM1_ESM.docx]

upporting information:

**Figure S1: Methylation level distribution for non-CpG sites (>0%).** Methylation level on the x-axis is divided in 10 bins. The y-axis indicates the proportion of total methylated Cs for each bin.


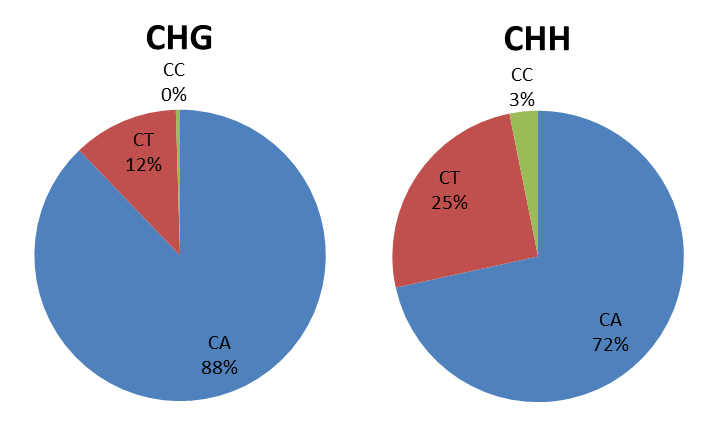


**Figure S2: non-CpG dinucleotide methylation preferences.** Figure shows that 88% of the CHG methylated (>10%) and 72% of the CHH methylated sites occur on CpA dinucleotide sites in brain.


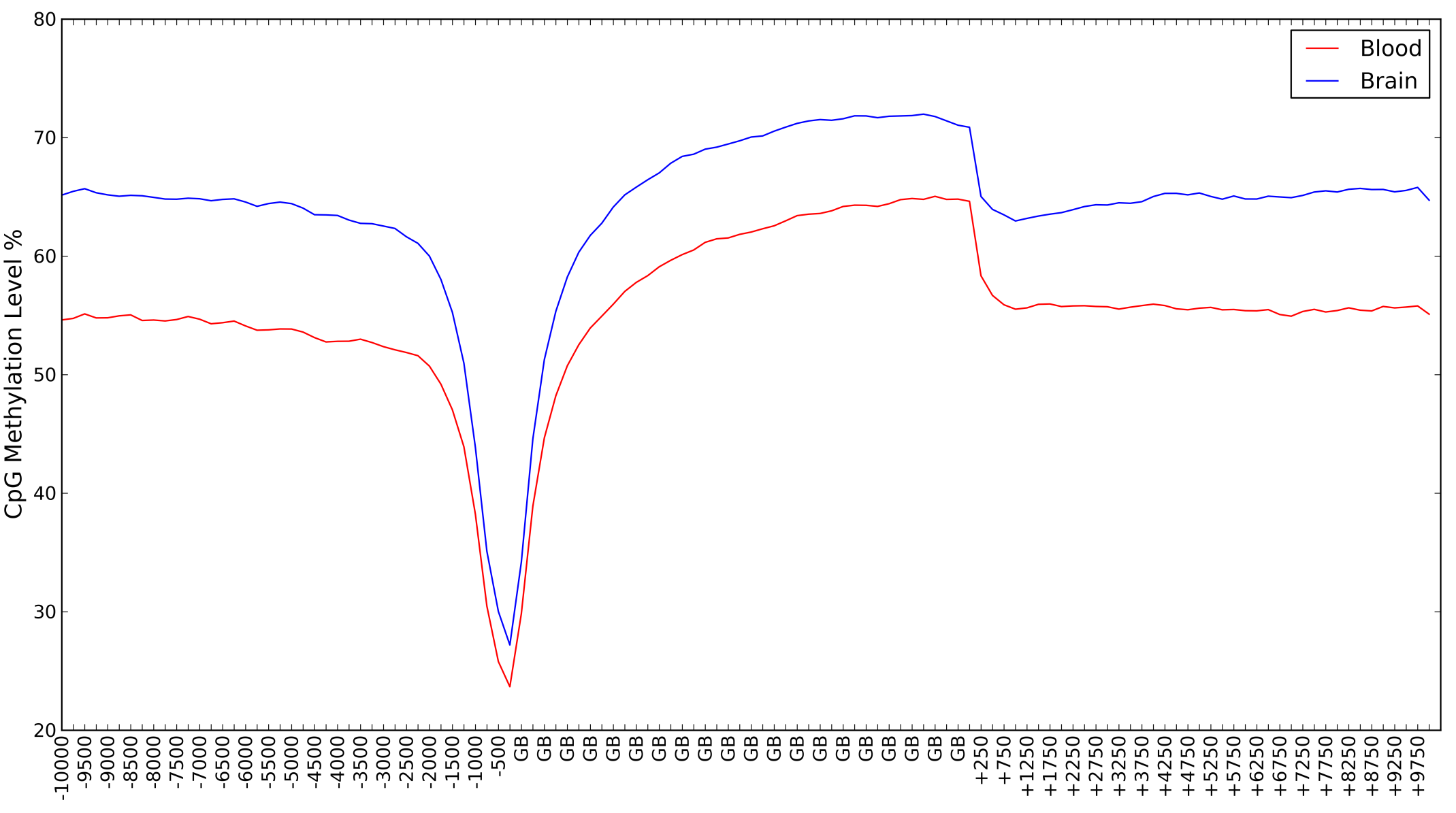


**Figure S3: CpG methylation level distribution in genes.** Genes are divided into 80 overlapping sliding windows, of which 20 windows in both up and downstream 10kb regions and 40 windows in the gene body. Figure shows decreased methylation near the transcription start site (TSS) and transcription termination site (TTS).


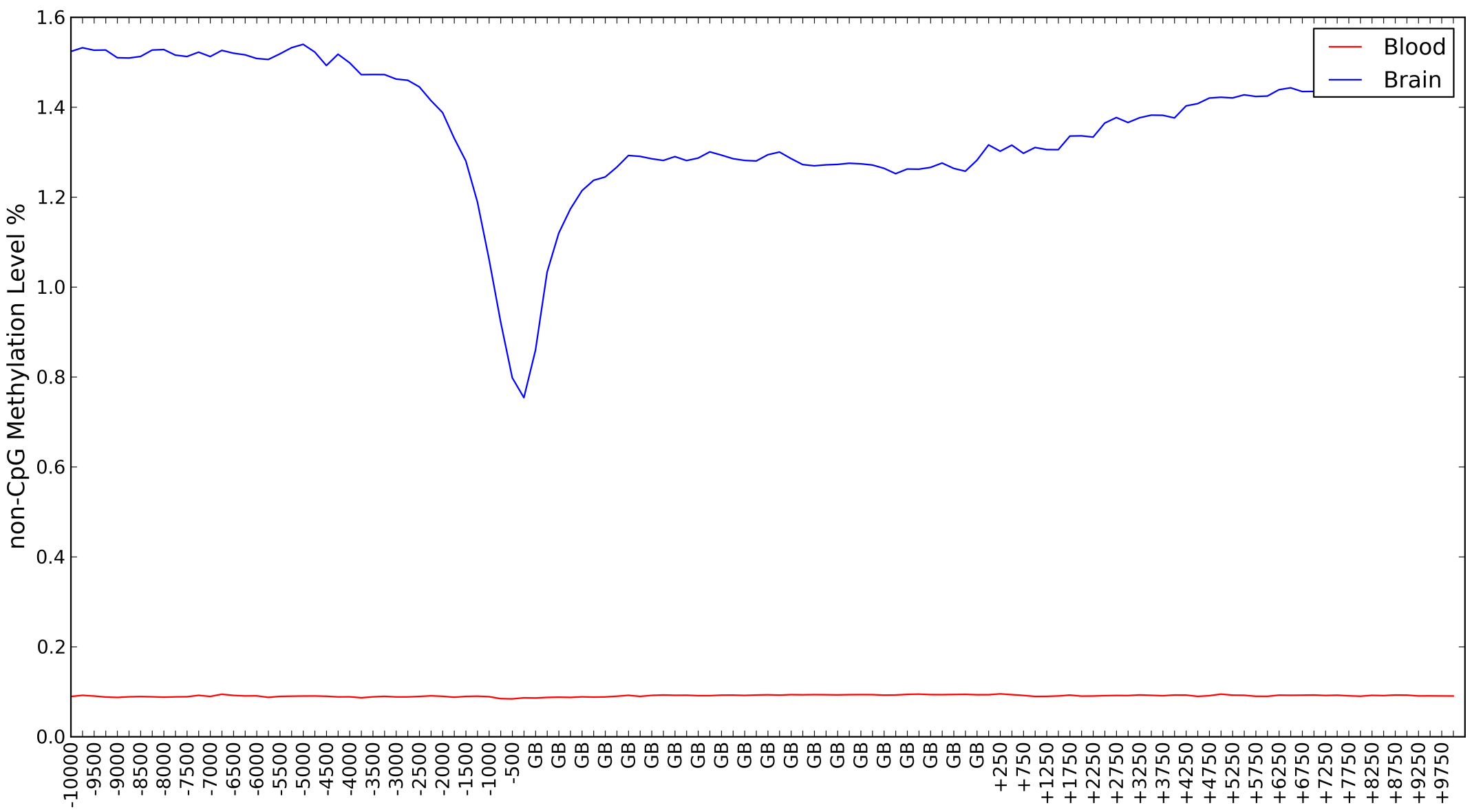


**Figure S4: non-CpG methylation level distribution in genes.** Genes are divided into 80 overlapping sliding windows including 20 windows in both up and downstream 10kb regions and 40 windows in the gene body. Figure shows decreased methylation near the transcription start site (TSS) in brain.


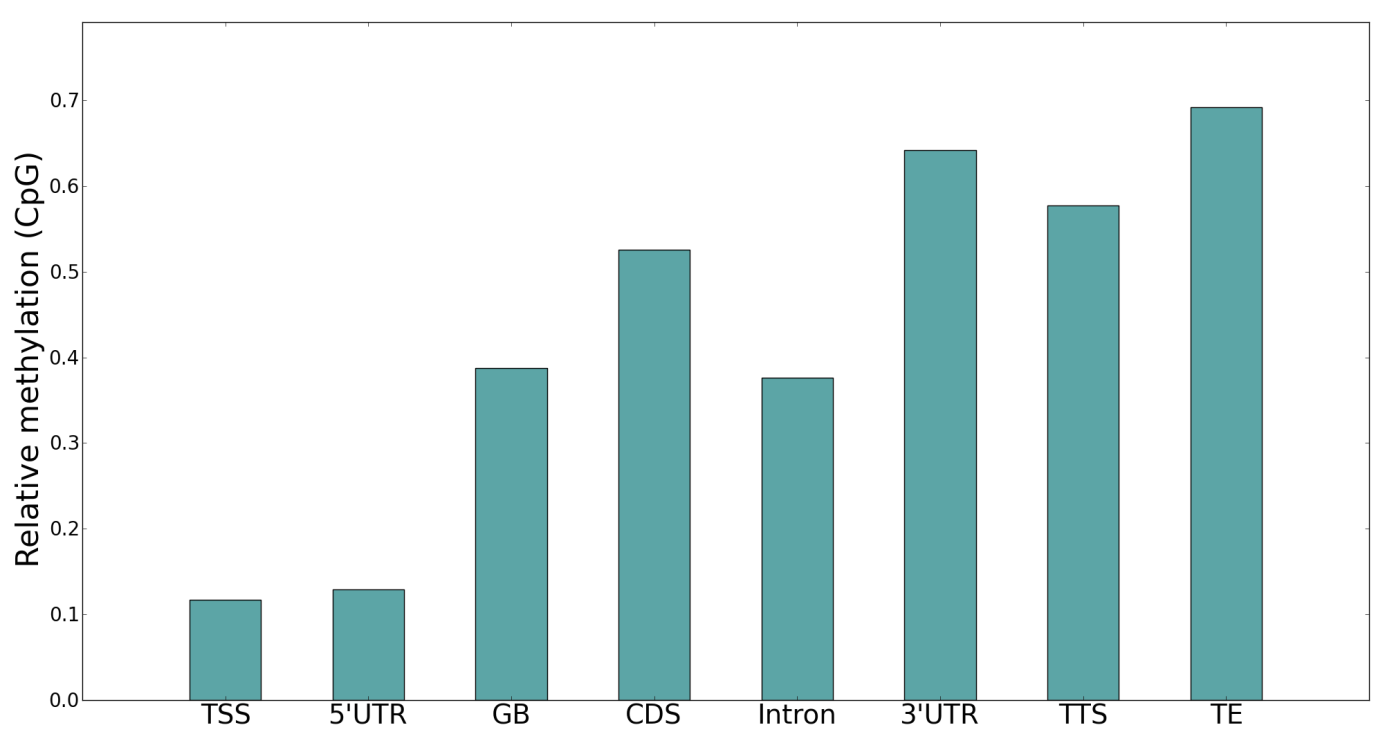


**Figure S5: Average CpG methylation in different gene partitions.** Figure shows average methylation over all CpG sites (blood and brain combined). Only genes with both UTRs annotated are included in this analysis. TSS and 5’UTR are hypomethylated while 3’UTR, CDS and TTS are largely hypermethylated.


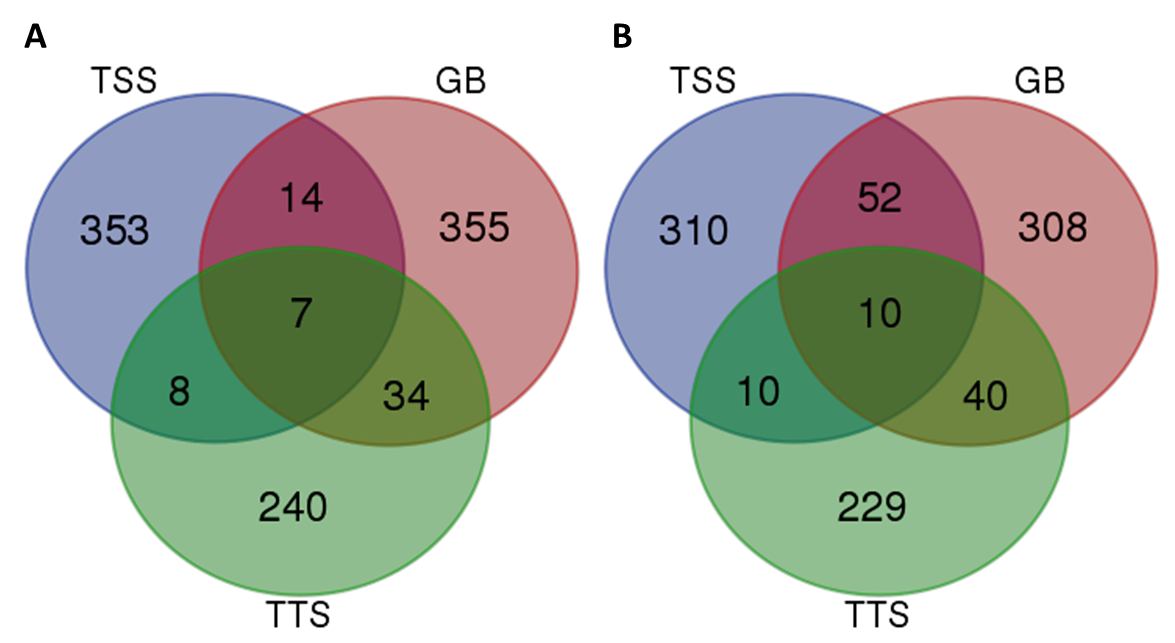


**Figure S6: The overlap for brain differentially hypo-methylated (A) and hyper-methylated (B) gene features.**


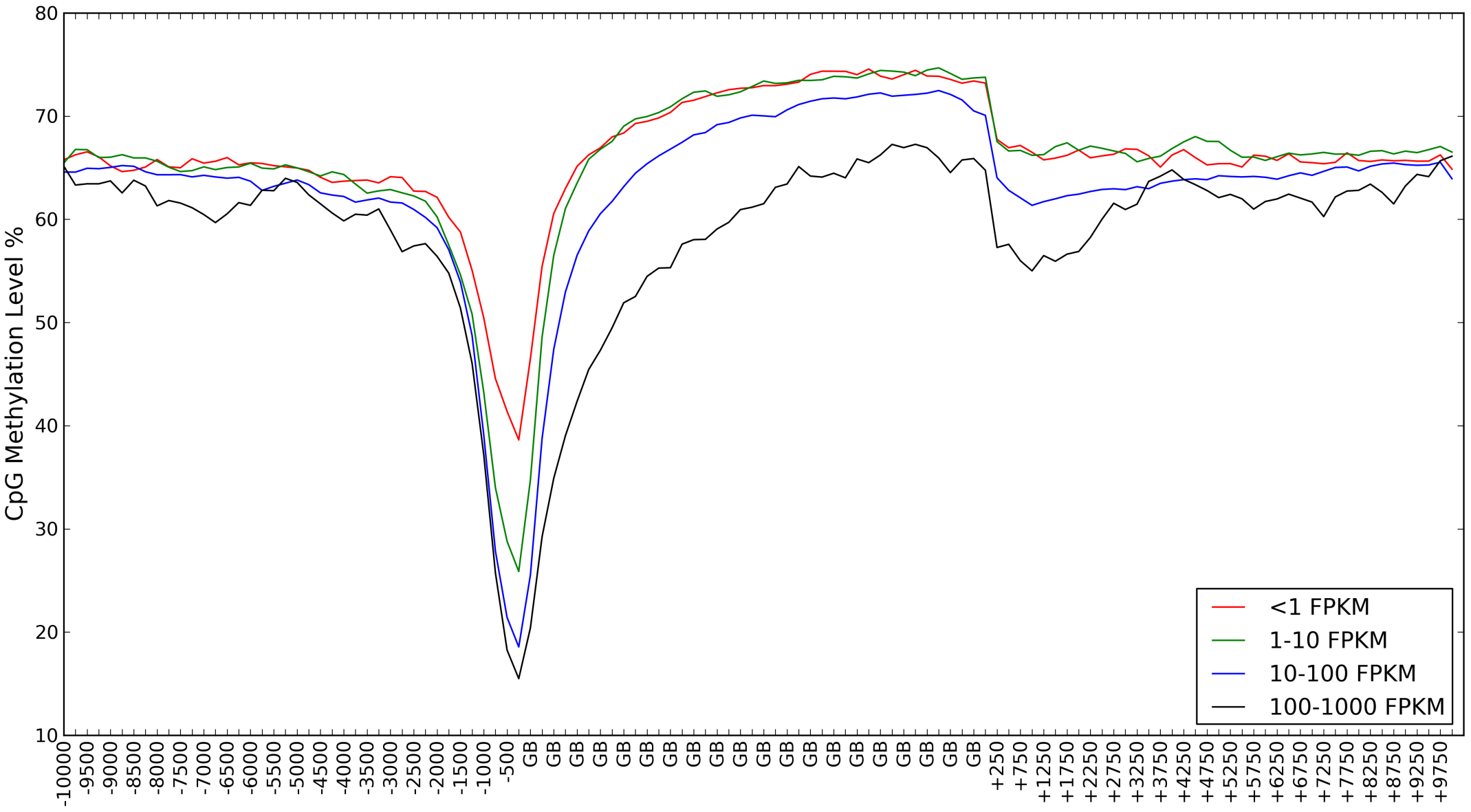


**Figure S7: CpG methylation in relation to gene expression in brain.** Highly expressed genes show lower methylation patterns over the entire gene length.

**Figure S8: Relative CpG methylation for CGIs divided over three genomic regional classes.** Promoter; 2kb upstream, 500 bp downstream of TSS. Intragenic; within the boundaries of a gene or completely overlapping. Intergenic; not annotated in genic regions.

**Figure S9: Relationship between non-CpG methylation level and gene length.** Figure shows a positive correlation for brain non-CpG methylation levels and gene length.


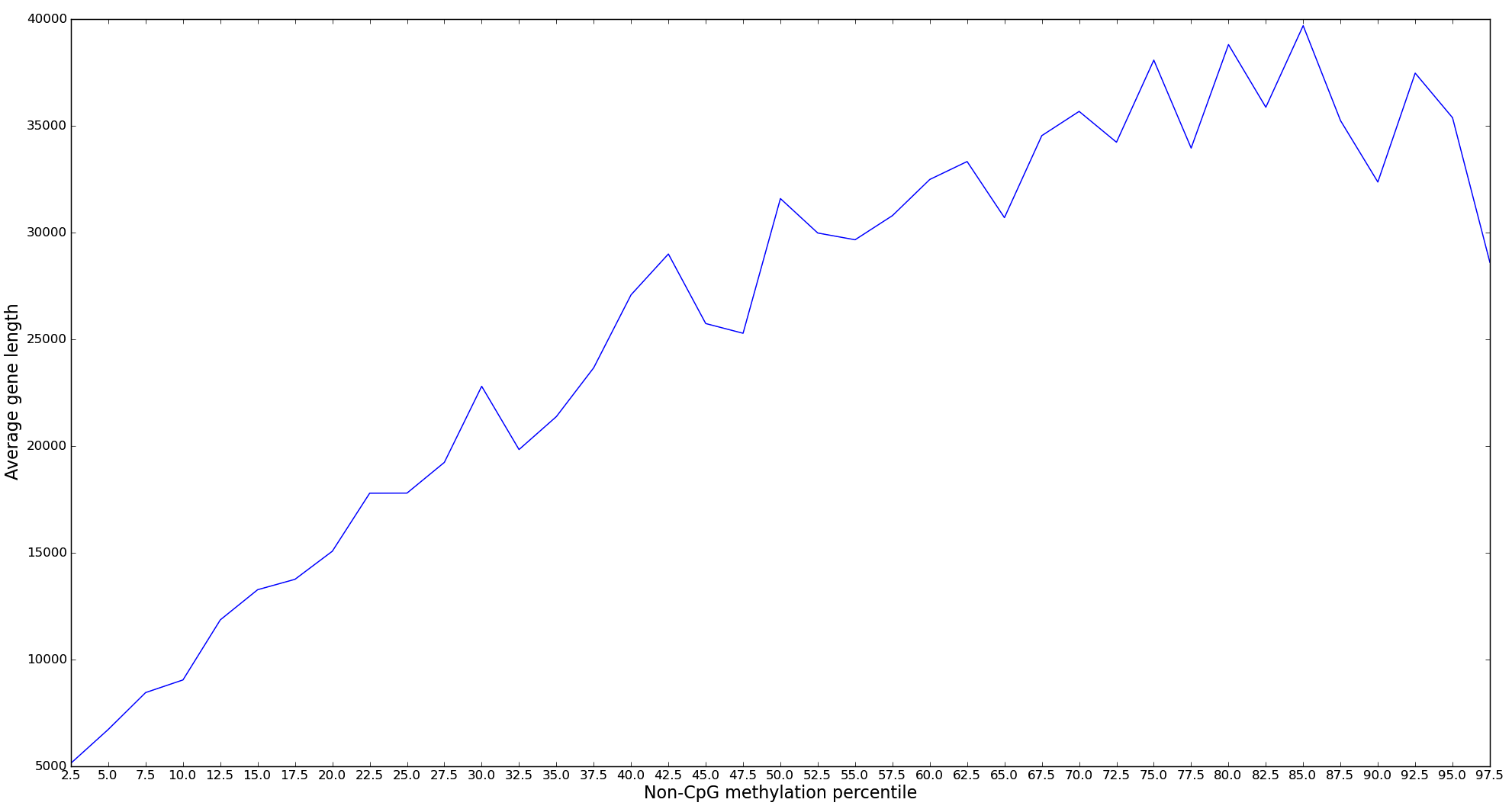


**Figure S10: Average gene length for 40 groups of percentiles of non-CpG methylated genes.** Genes were divided in 40 groups of percentiles based on their level of non-CpG methylation.


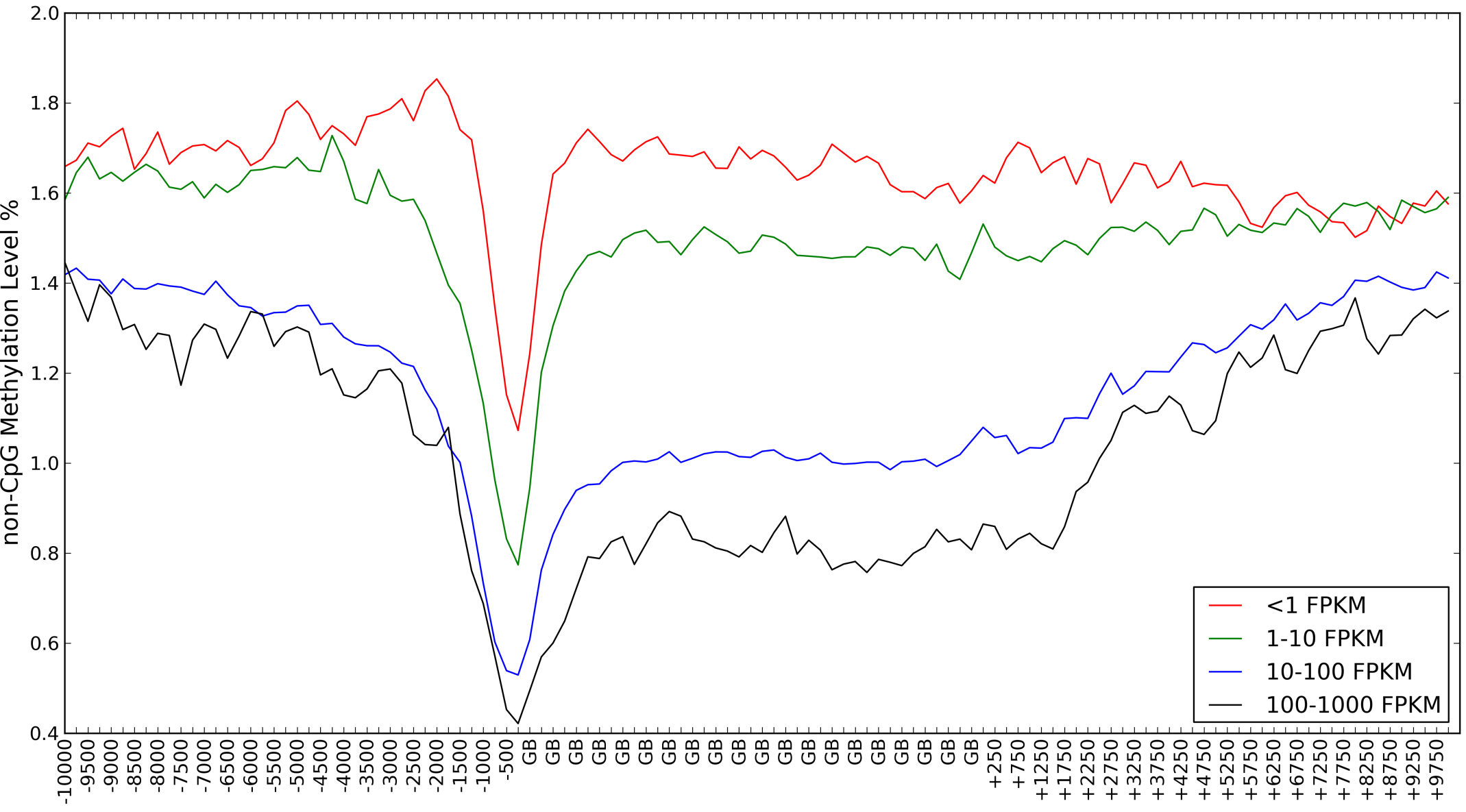


**Figure S11: Non-CpG methylation in relation to gene expression in brain.** Highly expressed genes show lower non-CpG methylation patterns over the entire gene length.


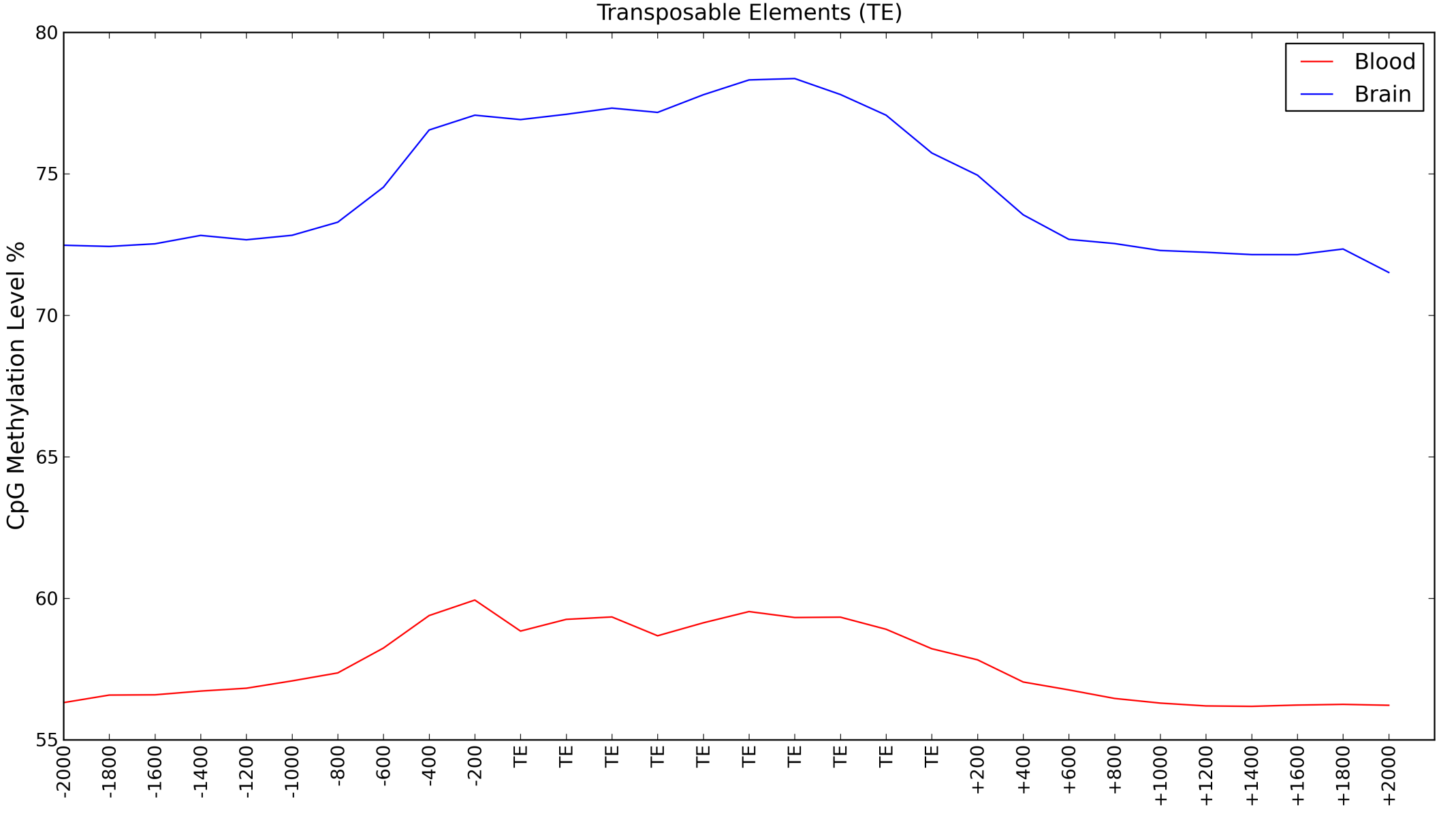


**Figure S12: CpG methylation level distribution in TEs and their 2kb flanking regions.** Figure shows increased methylation level in TE body.


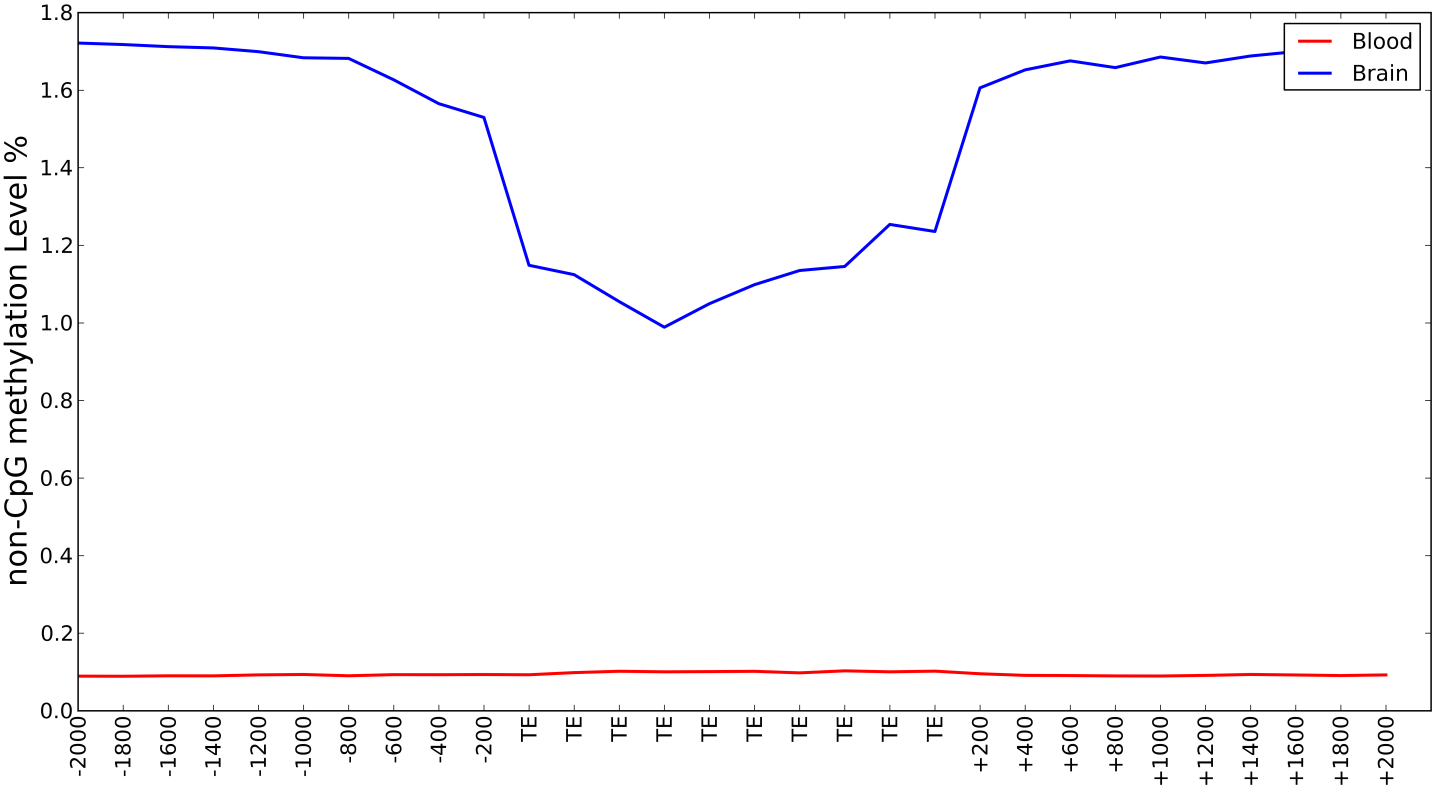


**Figure S13: Non-CpG methylation level distribution in TEs and their 2kb flanking regions.** Figure shows decreased non-CpG methylation level in TE body.


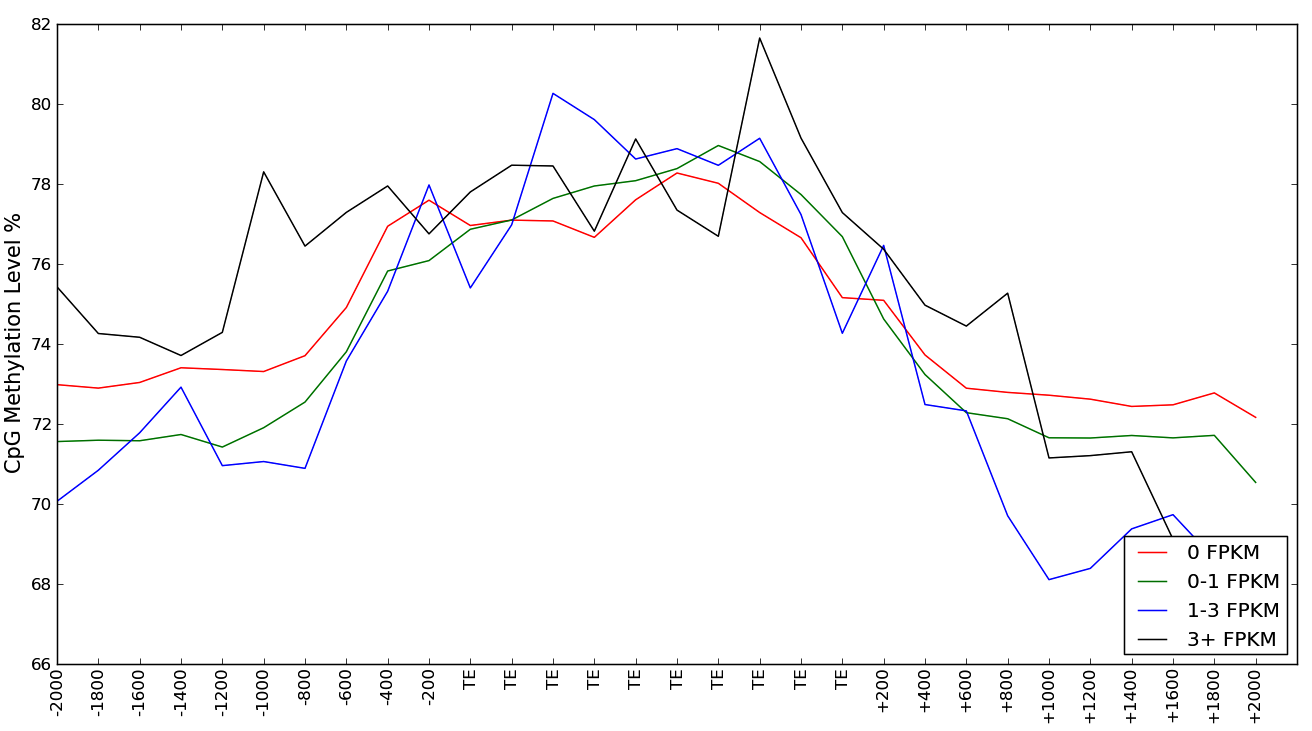


**Figure S14: CpG methylation in relation to TE expression in the brain.** Figure shows no correlation between TE expression and CpG methylation level.

.
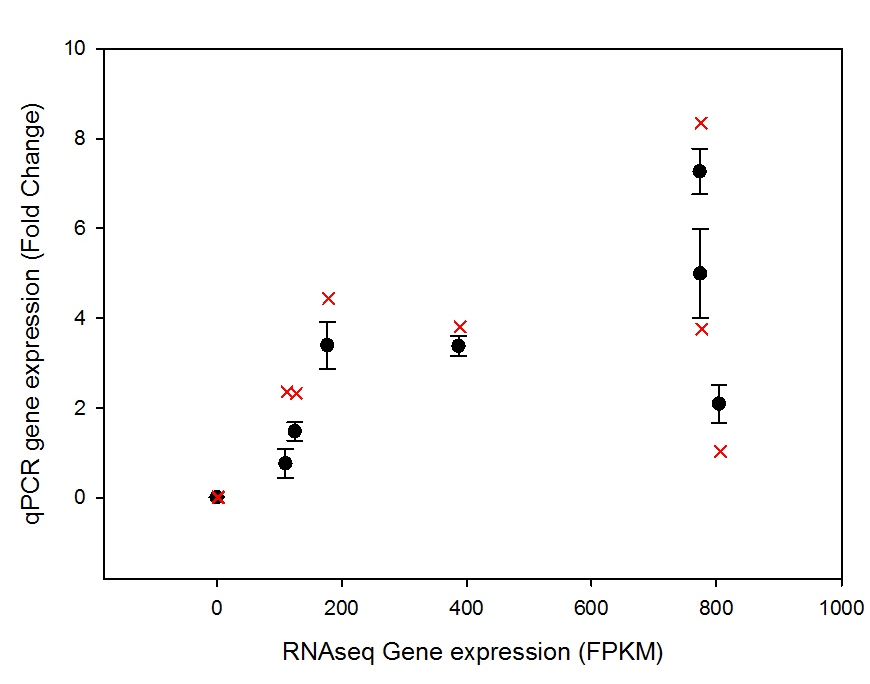


**Figure S15: Standardized gene expression from qPCR (Fold Change) as a function of the gene expression calculated from RNA-seq.** Data in fragments per kilobase of exons per million mapped reads (FPKM) for 11 genes relative to GADPH. Black circles indicate the mean value of 5 individuals. The reference individual used for WGBS and RNA-seq is indicated with red crosses. The four lowly expressed genes are overlapping in the figure.

**Figure S16: Whole genome bisulfite sequencing (WGBS) vs. Reduced representation bisulfite sequencing (RRBS) in blood.** 2,000 randomly selected methylated CpG sites ranging in methylation level between 0 and 100% in the WGBS sample were selected and compared with two RRBS samples. Figure shows strong correlation (Pearson r: 0.758 and 0.767) between WGBS and RRBS samples

**Table S1: Average and median gene expression in brain for genes associated with differentially methylated CGIs**.

| **Gene set** | **Average FPKM** | **Median FPKM** |
| --- | --- | --- |
| Promoter brain hypomethylated | 36.3 | 16.0 |
| Promoter brain hypermethylated | 13.9 | 1.5 |
| Intragenic brain hypomethylated | 50.2 | 23.7 |
| Intragenic brain hypermethylated | 23.5 | 6.6 |

**Table S2: Average and median gene expression levels for upper and lower 2.5% non-CpG methylated genes (brain)**.

| **Gene set** | **Average FPKM** | **Median FPKM** |
| --- | --- | --- |
| TSS lower 2.5% | 42.5 | 17.4 |
| Gene body lower 2.5% | 59.1 | 26.6 |
| TTS lower 2.5% | 37.3 | 16.6 |
| TSS upper 2.5% | 18.5 | 5.7 |
| Gene body upper 2.5% | 12.0 | 1.7 |
| TTS upper 2.5% | 15.1 | 5.7 |
| *Total gene set* | *28.1* | *11.3* |

**Table S3: Blast2GO gene ontology annotation.**

| **Subject species** | *Homo sapiens, Mus musculus, Gallus gallus* |
| --- | --- |
| **Input transcripts** | 16,424 |
| **Annotated** | 15,186 |
| **total GO annotations** | 247,144 |
| **GO/gene** | 16.27 |

**Table S4: Methylation profiles in two blood RRBS samples.**

| **Site** | **Sample** | **Covered sites** | **Average methylation level** | **Methylated sites (>10%)** |
| --- | --- | --- | --- | --- |
| **CpG** | RRBS1 | 2313208 | 23.39 | 678518 |
|  | RRBS2 | 2309957 | 21.74 | 674207 |
| **CHG** | RRBS1 | 2227429 | 0.33 | 5132 |
|  | RRBS2 | 2222074 | 0.38 | 5623 |
| **CHH** | RRBS1 | 3746455 | 0.32 | 9717 |
|  | RRBS2 | 3730539 | 0.37 | 10243 |

**Table S5: Primer information for the genes used for qPCR validation. Ensembl gene symbols are given for the great tit genes.**

| **Gene** | **Forward primer** | **Reverse primer** |
| --- | --- | --- |
| GAPDH | 5′-AGATGCGATGTCCAAGGCTC-3′ | 5′-AGAAGTCCACCCTGCCTGAT-3′ |
| BMAL1 | 5′-CGCTTCGTGGTGCTACAAAC-3′ | 5′-CCATCTGCTGCCCTGAGAAT-3′ |
| Cry1 | 5′-TCGCTTGTAAGTAAGAGGAGGC-3′ | 5′-AGCTGGAGTGGAGGTCATTG-3′ |
| Dio2 | 5′-TCCACACTTGCCACCAACAT-3′ | 5′-CAAACTGGGAGGAGAAGCCC-3′ |
| DrD4 | 5′-TTGGCTGGGCTACGTCAAC-3′ | 5′-AGCAGAAGACATGCAAGACT-3′ |
| GR | 5′-TCTCCATCCACAACCTCAGC-3′ | 5′-CACACGTCAGGACACCGTAG-3′ |
| HBMS | 5′-GGTCTTTGGCACGAACTTCC-3′ | 5′-CTGGGCTGAAGAGAATGGGC-3′ |
| LHR | 5′-AGGCGGATACACAACGATGC-3′ | 5′-CCTCAAGCCCGTAACTAGGC-3′ |
| MR | 5′-CTGCCCAGCCTGTCGATTA-3′ | 5′-CTCCTCGTGCATCCCTTTCA-3′ |
| PMM1 | 5′-TTGATGTCTTCCCAGAGGGC-3′ | 5′-GGGGTTGTCTCGTTCCCAAA-3′ |
| SERT | 5′-AGCTGACATTGCCCTTGCT-3′ | 5′-CTCATCTCCTCCTTCACGGC-3′ |
| StAR | 5′-TCTCAGCCCTTATGAACCCC-3′ | 5′-GTGCGGTGCTCCAAGAGAC-3′ |
